# Zfp36l1 Inhibits DNA Damage by Regulating p21-E2F1-Rad51 Signaling During Myogenic Differentiation

**DOI:** 10.3390/ijms27125319

**Published:** 2026-06-12

**Authors:** Yi Liu, Xiaoyu Jiang, Jingxin Sun, Luyao Wang, Jialong Li, Honglin Liu, Aiwen Jiang, Shenglong Wu, Wenbin Bao

**Affiliations:** 1 Key Laboratory for Animal Genetics, Breeding, Reproduction and Molecular Design, College of Animal Science and Technology, Yangzhou University, Yangzhou 225009, China; mx120240931@stu.yzu.edu.cn (Y.L.); 15252752609@163.com (J.S.); mx120230893@stu.yzu.edu.cn (L.W.); lijialong0756@163.com (J.L.); 008467@yzu.edu.cn (A.J.); 2 Department of Animal Genetics, Breeding and Reproduction, College of Animal Science and Technology, Nanjing Agricultural University, Nanjing 210095, China; mz120231591@stu.yzu.edu.cn (X.J.); liuhonglin@njau.edu.cn (H.L.); 3 Joint International Research Laboratory of Agriculture & Agri-Product Safety, Yangzhou University, Yangzhou 225009, China

**Keywords:** Zfp36l1, myogenic differentiation, DNA damage, p21, homologous recombination

## Abstract

Skeletal muscle differentiation relies on transient DNA strand breaks (DSBs), yet excessive DNA damage remains harmful to myogenic progression. The RNA-binding protein Zfp36l1 is expressed in skeletal muscle and contributes to muscle regeneration; nevertheless, its role in preserving genome stability during myogenic differentiation has not been defined. Here, we investigated the role and mechanism of Zfp36l1 in regulating DNA damage using C2C12 myoblast cells, combining loss- and gain-of-function assays, RNA-seq, and rescue experiments. The results revealed that Zfp36l1 expression is strongly induced during early myogenic differentiation, coinciding with the onset of physiological DSBs. Functional assays revealed that silencing Zfp36l1 aggravates DSB accumulation, reinforces G0/G1 cell cycle arrest, and promotes apoptosis, whereas Zfp36l1 overexpression attenuates these abnormalities. Transcriptomic profiling shows that Zfp36l1 knockdown impairs homologous recombination (HR)-mediated DNA repair by downregulating core repair factors, including *Rad51* and *Brca1*. Gene set enrichment analysis further confirms significant suppression of the HR-dependent DSB repair pathway. Mechanistically, Zfp36l1 regulates HR repair by suppressing p21 expression, thereby relieving inhibition of E2F1-mediated *Rad51* transcription. Co-silencing p21 restores *Rad51* expression and reduces DNA damage in Zfp36l1-knockdown cells. Collectively, these findings identify Zfp36l1 as an essential safeguard of genome stability during myogenic differentiation by balancing DNA damage levels through the p21-E2F1-Rad51 signaling axis, and provide new insights into the regulatory basis of muscle development and genomic instability-associated muscle diseases.

## 1. Introduction

Skeletal muscle represents approximately 40% of mammalian body weight and plays essential roles in locomotion, systemic metabolism, and energy homeostasis [1,2]. Disruptions in proper myogenic differentiation are strongly linked to various muscle disorders, including muscular atrophy, sarcopenia, and muscular dystrophy [3,4,5]. Therefore, deciphering the molecular networks that govern myogenic differentiation is critical for understanding muscle development and designing therapies for pathological conditions.

Myogenic differentiation proceeds through a tightly ordered sequence in which proliferating myoblasts exit the cell cycle irreversibly, undergo extensive morphological and biochemical remodeling, and ultimately fuse to form multinucleated myotubes [6,7,8]. Multiple biological processes, including autophagy and apoptosis, are precisely coordinated during this transition [5,9]. Notably, accumulating evidence, including our previous work, has demonstrated that transient DNA damage and apoptosis occur during skeletal muscle differentiation and participate in regulating myoblast fusion [5,10,11]. Researchers found that DNase activated by Caspase-3 (CASP3) facilitates myogenic differentiation via inducing DNA strand breaks (DSBs) [10,11]. However, unrepaired DNA damage resulting from a defective DNA damage response (DDR) triggers pathological apoptosis and arrests myogenic differentiation. Hence, identifying key regulators that maintain DNA damage within a physiological range is essential for sustaining myogenesis.

The DDR is a complex signaling cascade that detects and repairs various forms of DNA lesions, including double-strand breaks, single-strand breaks, and base damage [12]. This intricate network involves multiple sensors, transducers, and effector proteins that coordinate cell cycle arrest, DNA repair, and, if damage is irreparable, apoptosis or senescence. One well-characterized axis centers on Ataxia Telangiectasia Mutated (ATM) and Ataxia Telangiectasia and Rad3-related (ATR) kinases, as well as their downstream checkpoint kinase 1 (CHK1) and checkpoint kinase 2 (CHK2) signaling. This cascade further modulates the p53-p21 pathway to regulate cell cycle arrest and apoptosis [12]. RNA-binding proteins (RBPs) have recently emerged as important post-transcriptional regulators of DDR control and genome integrity maintenance [13]. The Zfp36/TTP family, comprising Zfp36, Zfp36l1, and Zfp36l2, regulates mRNA stability by binding to AU-rich elements in target transcripts, thereby modulating inflammation, senescence, and genome stability [14,15,16]. Zfp36l1 is widely expressed in multiple tissues, including skeletal muscle [17], and has been implicated in DNA repair in certain cellular contexts [18]. For instance, Zfp36l1 regulates non-homologous end joining (NHEJ) in osteosarcoma cells and safeguards genome stability in B cells under replication stress [18]. Despite these advances, whether Zfp36l1 regulates DNA damage repair and genome stability during myogenic differentiation remains unclear.

In this study, C2C12 myoblast cell line was adopted as the research model. Combining loss- and gain-of-function assays, RNA-seq, and rescue experiments, this study aimed to explore the biological function and underlying molecular mechanism of Zfp36l1 in modulating DNA damage homeostasis during myogenic differentiation. This work intends to deepen the understanding of muscle developmental regulation and offer theoretical reference for muscle disorders correlated with genomic instability.

## 2. Results

### 2.1. DNA Damage Accumulates During Early Stage of C2C12 Myogenic Differentiation

**Figure 1:**
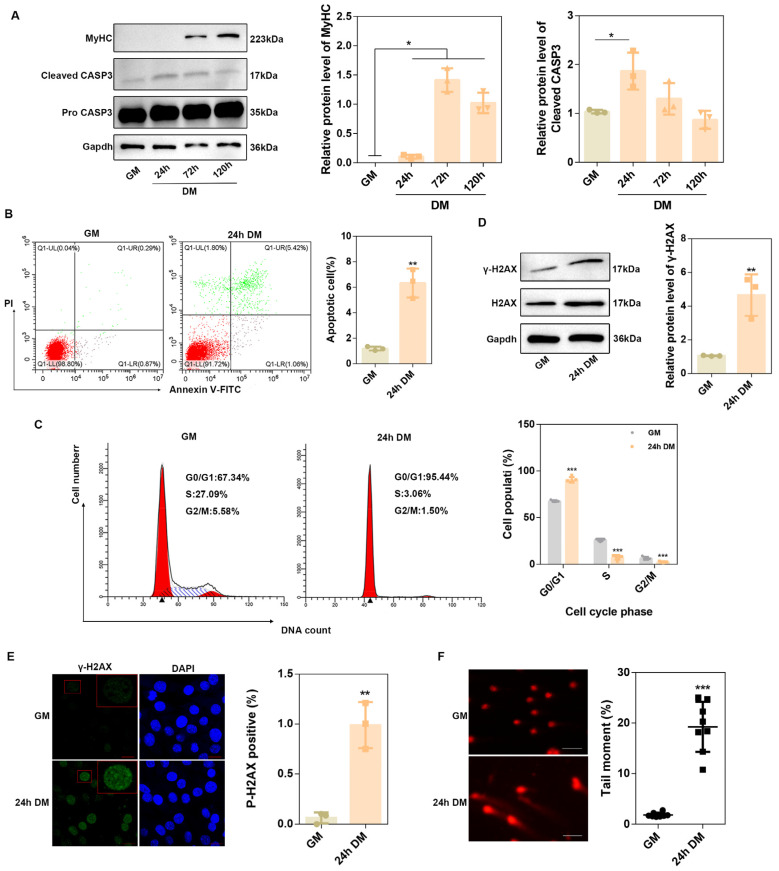
Analysis of DNA damage during C2C12 differentiation. (**A**) Western blotting detected protein levels of myosin heavy chain (MyHC) and cleaved CASP3 in C2C12 cells cultured in growth medium (GM) or differentiation medium (DM) for 0, 24, 72, and 120 h. Gapdh served as a loading control. (**B**) Apoptotic cell rates were determined by flow cytometry using Annexin V/PI staining at GM and 24 h DM. The percentages of apoptotic cells are presented in the bar graph. (**C**) Cell cycle distribution analyzed by flow cytometry at GM and 24 h DM. Representative cell cycle distribution and quantification of cell percentages in G0/G1, S, and G2/M phases are shown. (**D**) Western blot analysis of γ-H2AX, a marker of DNA double-strand breaks, at GM and 24 h DM. Gapdh served as a loading control. (**E**) Immunofluorescence staining of γ-H2AX (green) in C2C12 cells at GM and 24 h DM. Nuclei were counterstained with DAPI (blue). Scale bar, 20 μm. The red frame marks the magnified view of γ-H2AX staining. Representative images and quantification of γ-H2AX positive cells are shown. (**F**) DNA strand breaks assessed by the single-cell gel electrophoresis comet assay at GM and 24 h DM. Representative comet images (scale bar, 100 μm) and quantification of percentage of tail DNA are presented. Data are presented as mean ± SD. * *p* < 0.05, ** *p* < 0.01, *** *p* < 0.001.

To investigate the dynamics of DNA damage during myogenesis, we induced C2C12 myoblasts to differentiate by replacing growth medium (GM) with differentiation medium (DM) for up to 120 h. Myosin heavy chain (MyHC) is a marker of mature myotubes, and our results showed that its expression was significantly upregulated after differentiation induction, indicating the successful differentiation of C2C12 myoblast cells (Figure 1A). CASP3 serves as a key effector protease in apoptosis, and its activation requires proteolytic cleavage to generate the active cleaved CASP3 fragment [10]. Given that apoptosis accompanies myogenic differentiation [5], we examined cleaved CASP3 expression to assess potential apoptotic events during differentiation. Notably, the expression of Cleaved CASP3 was significantly increased as early as 24 h post-induction (Figure 1A). Correspondingly, the percentage of apoptotic cells was also markedly elevated (Figure 1B). Cell cycle analysis revealed an arrest at the G0/G1 phase, accompanied by a decreased proportion of cells in the S phase (Figure 1C).

We next evaluated DNA damage via γ-H2AX staining, a canonical marker for DNA double-strand breaks. Both Western blot (Figure 1D) and immunofluorescence (Figure 1E) analyses showed that γ-H2AX were significantly upregulated after 24 h of differentiation. Furthermore, we performed the comet assay. The percentage of tail DNA, a commonly used index, reflects the degree of DNA fragmentation; a higher percentage indicates more severe DNA damage [10]. Our results revealed a significant increase in the percentage of tail DNA upon differentiation induction (Figure 1F). Collectively, these results demonstrate that significant DNA damage accumulates in C2C12 cells at the early stage of myogenic differentiation.

### 2.2. Zfp36l1 Is Elevated During Skeletal Muscle Development

**Figure 2:**
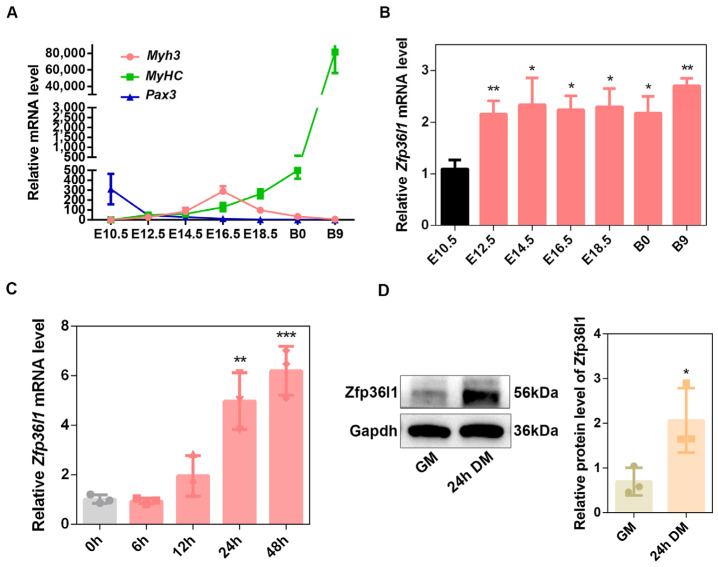
Analysis of Zfp36l1 expression during skeletal muscle development. (**A**) qRT-PCR measured the mRNA levels of embryonic myosin (*Myh3*), *MyHC*, and the progenitor cell marker *Pax3* during mouse embryonic development (E10.5 to E18.5) and at postnatal stages (birth, B0, and postnatal day 9, B9). Data are shown as relative expression normalized to *Gapdh*. (**B**) *Zfp36l1* mRNA levels at the indicated developmental stages (E10.5 to B9) were determined by qRT-PCR. (**C**) *Zfp36l1* mRNA levels were measured by qRT-PCR in C2C12 cells at 0, 6, 12, 24, and 48 h after differentiation induction. Data are shown as relative expression normalized to *Gapdh*. (**D**) Zfp36l1 protein levels were analyzed by Western blotting at GM and 24 h DM, with Gapdh as a loading control. Data are presented as mean ± SD. * *p* < 0.05, ** *p* < 0.01, *** *p* < 0.001.

To explore the potential role of Zfp36l1 in myogenesis, we first examined its expression pattern during skeletal muscle development in vivo. During embryonic development, both the expression of embryonic myosin (*Myh3*) and *MyHC* increased, whereas the progenitor cell marker *Pax3* decreased (Figure 2A), confirming the successful progression of skeletal muscle development. In this context, *Zfp36l1* expression was significantly upregulated during embryogenesis and remained high postnatally (Figure 2B). Consistently, in the in vitro C2C12 differentiation model, both the mRNA (Figure 2C) and protein (Figure 2D) levels of Zfp36l1 were robustly increased as early as 24 h after differentiation. This temporal correlation with the onset of DNA damage during early differentiation (Figure 1) suggests a potential role for Zfp36l1 in this process.

### 2.3. Zfp36l1 Knockdown Exacerbates DNA Damage Levels and Cell Apoptosis

**Figure 3:**
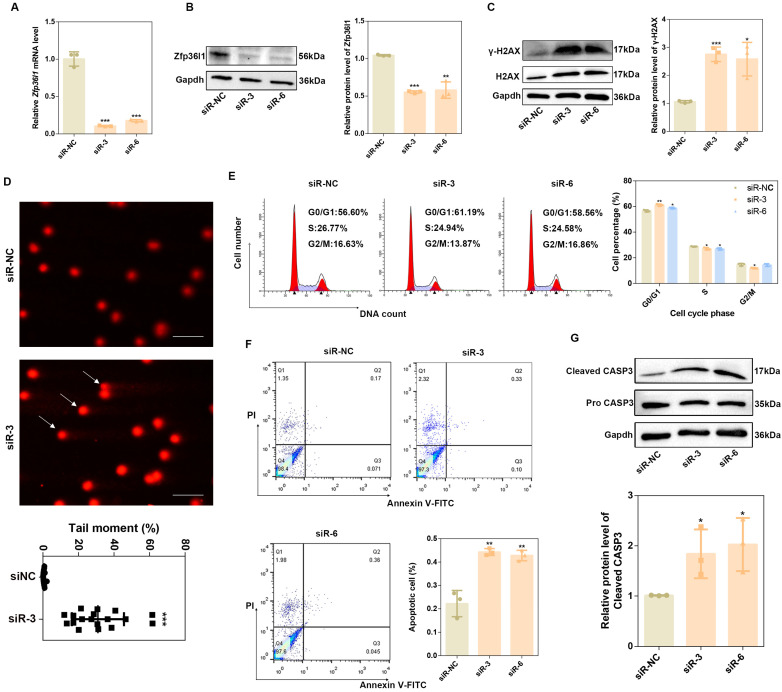
Analysis of DNA damage after Zfp36l1 knockdown. (**A**) *Zfp36l1* mRNA levels measured by qRT-PCR in C2C12 cells transfected with control siRNA (siNC) or Zfp36l1 siRNAs (siR-3 and siR-6). Data are shown as relative expression normalized to *Gapdh*. (**B**) Zfp36l1 protein levels measured by Western blot analysis in C2C12 cells transfected with siNC or Zfp36l1 siRNAs (siR-3 and siR-6). Gapdh served as a loading control. (**C**) Western blot analysis of γ-H2AX protein levels following Zfp36l1 knockdown. Gapdh served as a loading control. (**D**) Comet assay showing representative comet images and quantification of tail DNA percentage in siNC and siZfp36l1 groups. Scale bar, 100 μm. Arrows indicate fragmented cell nuclei. (**E**) Flow cytometry analysis of cell cycle distribution showing percentages of cells in G0/G1, S, and G2/M phases following Zfp36l1 knockdown. (**F**) Flow cytometry analysis of apoptotic cell percentage in siNC and siZfp36l1 groups. (**G**) Western blot analysis of cleaved CASP3 protein levels following Zfp36l1 knockdown. Gapdh served as a loading control. Data are presented as mean ± SD. * *p* < 0.05, ** *p* < 0.01, *** *p* < 0.001.

Given the concurrent increase in both DSB markers and Zfp36l1 expression during early differentiation, we investigated whether Zfp36l1 regulates DNA damage responses. Transfection with Zfp36l1 siRNAs (siR-3 and siR-6) effectively knocked down the mRNA (Figure 3A) and protein of Zfp36l1 expression in C2C12 cells (Figure 3B). Compared to the control, Zfp36l1 knockdown significantly upregulated the γ-H2AX protein levels (Figure 3C). Consistently, the percentage of tail DNA was significantly increased after Zfp36l1 knockdown (Figure 3D), indicating exacerbated DNA damage. This was accompanied by a pronounced G0/G1 cell cycle arrest (Figure 3E) and increased apoptotic rate (Figure 3F), and elevated Cleaved CASP3 levels (Figure 3G). These results suggest that loss of Zfp36l1 promotes DNA damage accumulation and apoptosis during differentiation.

### 2.4. Zfp36l1 Overexpression Alleviates DNA Damage and Inhibits Cell Apoptosis

**Figure 4:**
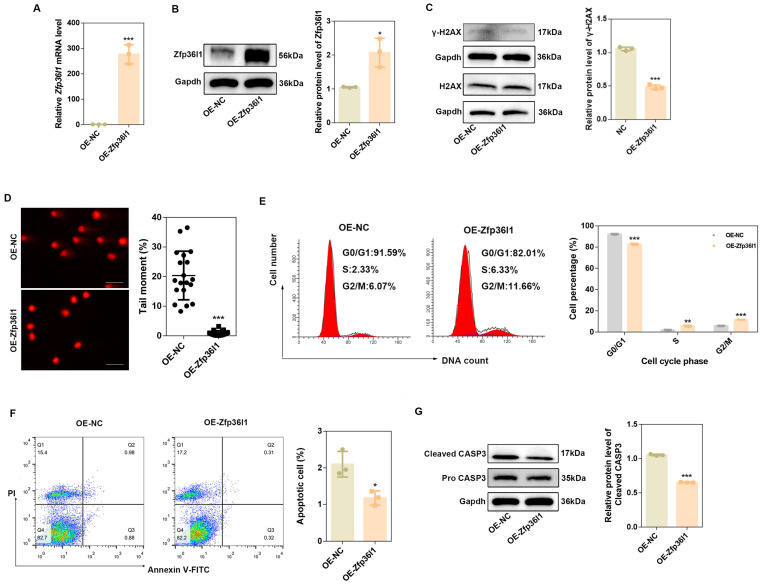
Analysis of DNA damage after Zfp36l1 overexpresison. (**A**) *Zfp36l1* mRNA levels measured by qRT-PCR in C2C12 cells transfected with OE-NC or OE-Zfp36l1. Data are shown as relative expression normalized to *Gapdh*. (**B**) Zfp36l1 protein levels measured by Western blot analysis after transfection with OE-NC or OE-Zfp36l1. Gapdh was used as a loading control. (**C**) Western blot analysis of γ-H2AX protein levels following Zfp36l1 overexpression. Gapdh was used as a loading control. (**D**) Comet assay showing representative comet images and quantification of tail DNA percentage in OE-NC and OE-Zfp36l1 groups. Scale bar, 100 μm. (**E**) Flow cytometry analysis of cell cycle distribution showing percentages of cells in G0/G1, S, and G2/M phases following Zfp36l1 overexpression. (**F**) Flow cytometry analysis of apoptotic cell percentage after transfection with OE-NC or OE-Zfp36l1. (**G**) Western blot analysis of cleaved CASP3 protein levels following Zfp36l1 overexpression. Gapdh served as a loading control. Data are presented as mean ± SD. * *p* < 0.05, ** *p* < 0.01, *** *p* < 0.001.

Consistent with the above results, the mRNA (Figure 4A) and protein levels (Figure 4B) of Zfp36l1 were significantly increased after OE-Zfp36l1 transfection. In addition, Zfp36l1 overexpression significantly reduced the protein levels of γ-H2AX (Figure 4C) and the percentage of tail DNA (Figure 4D). Cell cycle analysis showed that Zfp36l1 overexpression significantly decreased the percentage of cells in G0/G1 phase and increased the proportion in S phase (Figure 4E). Both the apoptotic rate of C2C12 cells (Figure 4F) and the protein level of Cleaved CASP3 (Figure 4G) were markedly decreased after OE-Zfp36l1 transfection. These results suggest that Zfp36l1 alleviates DNA damage accumulation and suppresses apoptosis during C2C12 differentiation.

### 2.5. RNA-Seq Analysis Reveals That Zfp36l1 Knockdown Impairs the Homologous Recombination Repair Pathway

**Figure 5:**
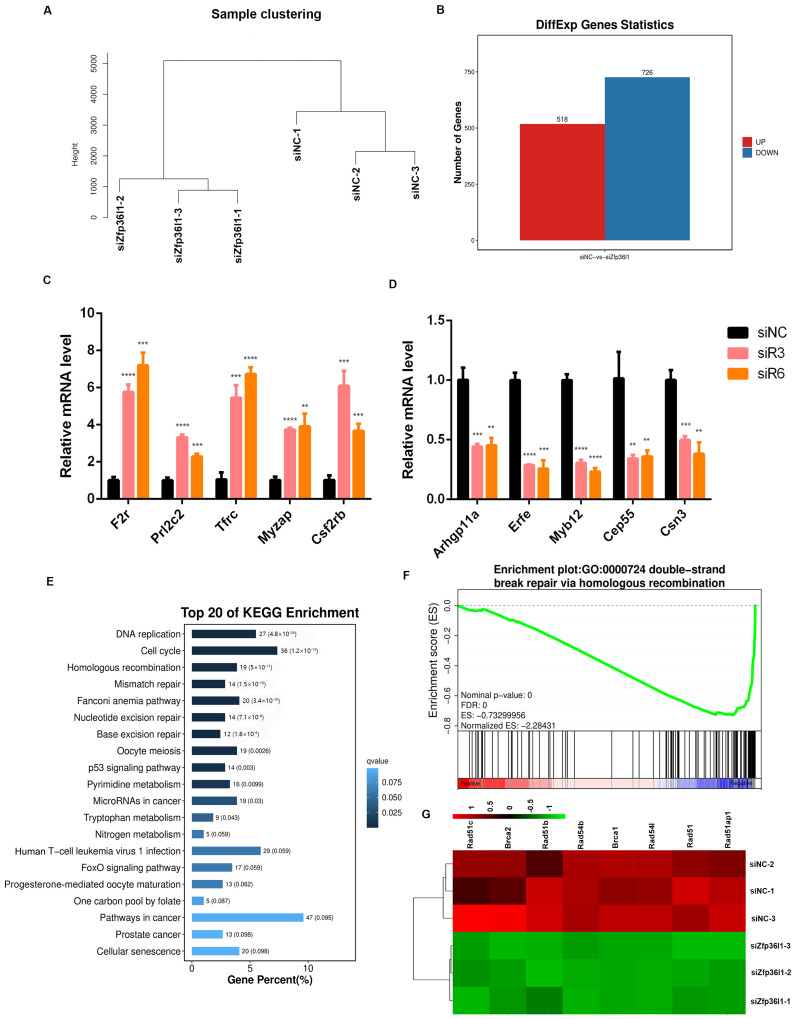
RNA-seq analysis after Zfp36l1 knockdown. (**A**) Hierarchical clustering of gene expression profiles between siNC and siZfp36l1 groups. (**B**) Volcano plot of gene expression differences between siNC and siZfp36l1 groups. (**C**,**D**) qRT-PCR validation of RNA-seq data for selected upregulated (**C**) and downregulated (**D**) genes. Data are shown as relative expression normalized to Gapdh. (**E**) KEGG pathway enrichment analysis of differentially expressed genes. (**F**) GSEA of the “double-strand break repair via homologous recombination” pathway. (**G**) Heatmap of key homologous recombination repair genes. Data are presented as mean ± SD. ** *p* < 0.01, *** *p* < 0.001, **** *p* < 0.0001.

To further explore the role of Zfp36l1, RNA-seq analysis was performed in C2C12 cells transfected with siZfp36l1. The dendrogram showed that all siZfp36l1 samples clustered together and were clearly separated from the siNC group (Figure 5A), indicating that Zfp36l1 knockdown markedly altered the gene expression profile of C2C12 cells. Differential expression analysis identified 518 upregulated and 726 downregulated differentially expressed genes (DEGs) in the siZfp36l1 group compared with the siNC group (Figure 5B and Table S4). Then, we randomly selected 5 upregulated genes (Figure 5C) and 5 downregulated genes (Figure 5D) for qRT-PCR to validate the RNA-seq data. Their expressions were consistent with the RNA-seq data (Figure 5C,D), verifying the reliability of the transcriptomic analysis. KEGG analysis for DEGs revealed that the “DNA replication”, “cell cycle”, “p53 signaling pathway” and “FoxO1 signaling pathway” were significantly enriched (Figure 5E), supporting the G0/G1 cell cycle arrest and apoptosis observed in Figure 3. Notably, the “homologous recombination” pathways were also enriched in KEGG analysis (Figure 5E), and GSEA results further confirmed that the “double-strand break repair via homologous recombination” pathway was markedly inhibited after Zfp36l1 knockdown (Figure 5F). Meanwhile, the key genes regulating homologous recombination repair (HRR), including *Rad51*, *Brca1*, *Brca2*, *Rad51b*, *Rad51c*, *Rad54b*, *Rad54l* and *Rad51ap1*, were all significantly decreased after Zfp36l1 knockdown (Figure 5G). In addition, qRT-PCR analysis further confirmed that the expression of several DDR-related genes is regulated by Zfp36l1 (Figure S1). These results suggest that knockdown of Zfp36l1 inhibits HHR, which exacerbates the accumulation of DNA damage.

### 2.6. Zfp36l1 Modulates Homologous Recombination Repair by Regulating p21-E2F1-Rad51 Signaling

**Figure 6:**
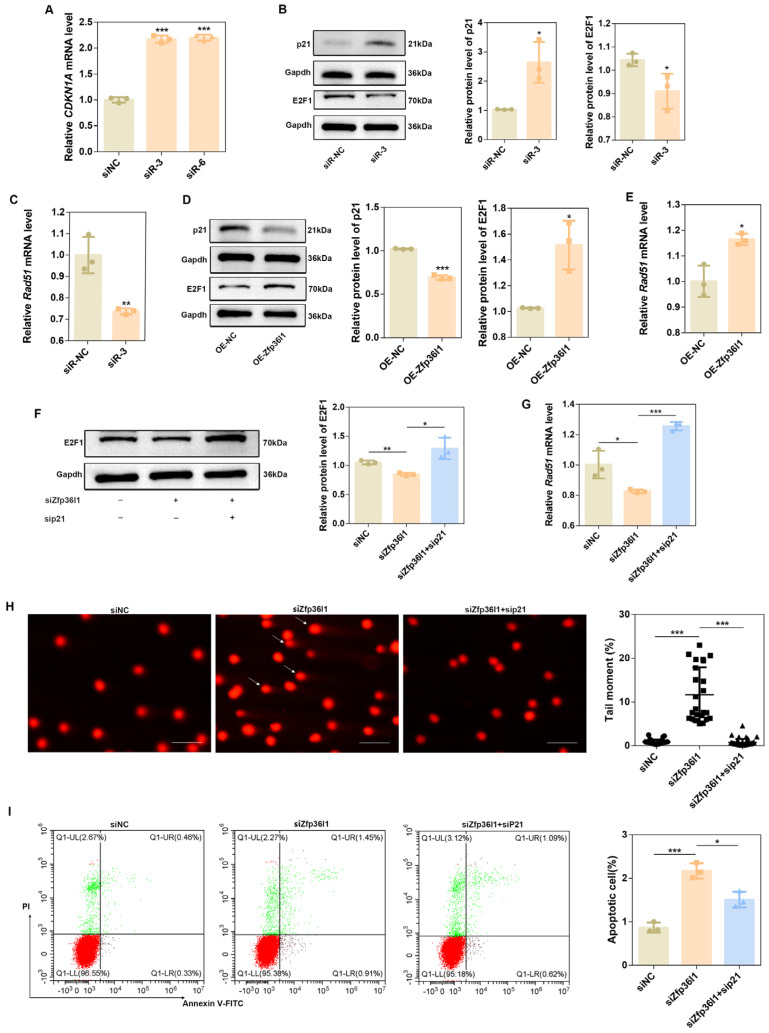
Rescue effect of p21 knockdown on DNA damage induced by Zfp36l1 deficiency. (**A**) qRT-PCR analysis of *CDKN1A* levels after Zfp36l1 knockdown. Data are shown as relative expression normalized to Gapdh. (**B**) Western blot of p21 and E2F1 protein levels following Zfp36l1 knockdown. Gapdh served as a loading control. (**C**) qRT-PCR analysis of *Rad51* mRNA levels following Zfp36l1 knockdown. Data are shown as relative expression normalized to Gapdh. (**D**) Western blot of p21 and E2F1 protein levels following Zfp36l1 overexpression. Gapdh served as a loading control. (**E**) qRT-PCR analysis of *Rad51* mRNA levels after Zfp36l1 overexpression. Data are shown as relative expression normalized to Gapdh. (**F**) Western blot analysis of E2F1 protein levels in cells with combined knockdown of Zfp36l1 and p21. Gapdh served as a loading control. (**G**) qRT-PCR analysis of *Rad51* mRNA levels in cells with combined knockdown of Zfp36l1 and p21. Data are shown as relative expression normalized to *Gapdh*. (**H**) Comet assay showing representative comet images and quantification of tail DNA percentage in cells with combined knockdown of Zfp36l1 and p21. Scale bar, 100 μm. Arrows indicate fragmented cell nuclei. (**I**) Flow cytometry analysis of apoptotic cell percentage in cells with combined knockdown of Zfp36l1 and p21. Data are mean ± SD. * *p* < 0.05, ** *p* < 0.01, *** *p* < 0.001.

CDKN1A, which encodes p21, is a well-characterized direct target gene of Zfp36l1 [19,20]. Proper regulation of p21 is critical for maintaining genome stability [21], partially through modulating E2F1-dependent *Rad51* transcription [22]. As shown in Figure 6A, Zfp36l1 knockdown significantly increased *CDKN1A* mRNA levels (*p* < 0.001). Notably, CDKN1A was also identified as a differentially expressed gene in our RNA-seq dataset (Table S4). Based on these findings, we hypothesized that Zfp36l1 mediates DNA damage repair via the p21-E2F1-Rad51 signaling pathway.

Western blot analysis showed that Zfp36l1 knockdown upregulated p21 protein levels while reducing E2F1 expression (Figure 6B). Consistently, *Rad51* mRNA levels were markedly reduced upon Zfp36l1 knockdown (Figure 6C). In contrast, Zfp36l1 overexpression decreased p21 and increased E2F1 protein abundance (Figure 6D), and elevated *Rad51* mRNA levels (Figure 6E), which was consistent with our transcriptomic results (Table S4 and Figure 5G).

Subsequently, we selected the siRNA with the highest knockdown efficiency against p21 (Figure S2A) and performed rescue experiments by dual silencing of p21 and Zfp36l1. Compared with the single Zfp36l1 knockdown group, co-knockdown of Zfp36l1 and p21 effectively restored E2F1 protein levels (Figure 6F) and *Rad51* mRNA expression (Figure 6G). Meanwhile, the elevated tail DNA percentage (Figure 6H) was also prominently rescued. Cell cycle analysis further showed that co-knockdown of Zfp36l1 and CDKN1A significantly decreased the percentage of cells in G0/G1 phase and increased the proportion in S phase compared with the single Zfp36l1 knockdown group (Figure S2B). Compared with the single Zfp36l1 knockdown group, co-knockdown of Zfp36l1 and p21 effectively reduced apoptotic rates (Figure 6I). Collectively, these results demonstrate that Zfp36l1 modulates DNA damage and homologous recombination repair through the p21–E2F1–Rad51 regulatory axis.

## 3. Discussion

Maintenance of genome stability is essential for proper cell fate determination, yet it is constantly challenged by endogenous and exogenous genotoxic stresses [23,24,25]. Among various DNA lesions, DNA double-strand breaks are the most cytotoxic [26]. Inefficient or inaccurate DSB repair can lead to genomic instability, malignant transformation, cellular senescence, or apoptotic cell death [27]. To counteract genotoxic stress, eukaryotic cells have evolved a sophisticated and tightly regulated DNA damage response network encompassing damage sensing, signal transduction, and repair pathways [28]. Homologous recombination represents a high-fidelity DSB repair pathway that is critical for preserving genome integrity during cell differentiation and proliferation [29].

During myogenic differentiation, we observed prominent cell cycle arrest and adaptive apoptotic characteristics accompanied by DSB occurrence (Figure 1). In line with our previous studies [9], moderate apoptosis facilitates cell differentiation via intercellular communication, whereas excessive apoptosis reduces cell viability and impairs myocyte fusion and myotube formation [5]. These findings underscore the necessity of tight regulatory mechanisms to maintain balanced DNA damage levels during myogenesis. An intriguing observation was that γ-H2AX exhibited pan-nuclear staining in C2C12 cells upon differentiation induction, and this phenotype is consistent with previous report [10]. This widespread DNA damage is likely associated with replication stress induced by myogenic differentiation.

Zfp36l1 has been implicated in cell cycle regulation, apoptosis, and stress responses [30,31]. Previous studies reported that Zfp36l1 suppresses hypoxic signaling and cell cycle progression in cancer cells and regulates senescence by modulating senescence-associated secretory phenotype (SASP) factors [30]. As a downstream effector of the mTOR signaling pathway, Zfp36l1 suppresses cellular senescence by degrading transcripts encoding numerous SASP components [32]. In skeletal muscle, combined deletion of Zfp36l1 and Zfp36l2 affects satellite cell maintenance and muscle regeneration [17]. However, the individual function of Zfp36l1 in myogenic differentiation and genome stability has not been previously illustrated. Our study demonstrates that Zfp36l1 expression is strongly induced during early myogenic differentiation (Figure 2). Moreover, its upregulation exhibited temporal coincidence with the appearance of DNA damage, suggesting that it may participate in the DNA damage response during myogenic differentiation.

Our loss- and gain-of-function experiments provide compelling evidence that Zfp36l1 plays an essential role in alleviating DNA damage during myogenesis. Knockdown of Zfp36l1 aggravated DSB accumulation, induced G0/G1 cell cycle arrest, and promoted apoptosis (Figure 3), whereas Zfp36l1 overexpression exerted cytoprotective effects (Figure 4). Transcriptomic analysis revealed that Zfp36l1 deficiency impairs the HR repair pathway, accompanied by significantly reduced expression of core HR genes including Rad51, Brca1, and several Rad51 paralogs (Figure 5). This further explains that the exacerbation of DNA damage following Zfp36l1 knockdown is attributable to impaired repair capacity. Zhang et al. reported that downregulation of ZFP36L1 promotes methotrexate resistance by enhancing NHEJ-mediated DNA repair [18]. In contrast to that previous finding, our results reveal two key roles of Zfp36l1: first, Zfp36l1 acts as a key regulator of HR repair; second, Zfp36l1 remains continuously highly expressed from the early stage of myogenic differentiation through to mature myotubes. Since mature myotubes are terminally differentiated and lack proliferative capacity, the high expression of Zfp36l1 in these cells may shift toward regulating NHEJ repair—a hypothesis that warrants further investigation.

p21 exerts dual functions in DNA damage repair. It induces cell cycle arrest to allow time for repair [33] while also directly facilitating homologous recombination repair by interacting with key factors such as PCNA [34,35]. Nevertheless, excessive p21 can suppress Rad51 expression via E2F1 inhibition, thereby compromising repair efficiency and promoting genomic instability [22]. As a well-identified target gene of Zfp36l1 [19], p21 expression was also regulated by Zfp36l1 in this study; rescue experiments further confirmed that Zfp36l1 modulates HR repair by preventing excessive p21 accumulation that would otherwise inhibit E2F1-dependent Rad51 transcription (Figure 6). This axis ensures sufficient HR capacity during myogenic differentiation, when cells undergo physiological replicative stress and DSB formation.

Although this study demonstrates that Zfp36l1 maintains genome stability during myogenic differentiation via the p21-E2F1-Rad51 axis, several limitations exist. First, our conclusions are drawn solely from C2C12 cell experiments, and in vivo evidence from muscle-specific Zfp36l1 knockout mice is needed for validation. Second, we mainly focused on homologous recombination repair; the roles of non-homologous end joining and other DNA repair pathways regulated by Zfp36l1 remain to be clarified. Third, further clinical and mechanistic studies are required to clarify whether Zfp36l1-mediated DNA damage regulation is implicated in muscular dystrophy, sarcopenia and other muscle disorders. In future studies, we will establish muscle-specific Zfp36l1 transgenic and knockout mouse models, explore its crosstalk with other DNA repair pathways, and verify its clinical relevance in muscle diseases.

## 4. Materials and Methods

### 4.1. Reagents

Cell Cycle Assay Kit (Cat No. KGA9101-50) and Comet Electrophoresis Kit (Cat No. KGA1303-50) were purchased from KeyGEN Biotech (Nanjing, Jiangsu, China). Annexin V/PI Apoptosis Detection Kit (Cat No. A211-01) was purchased from Vazyme (Nanjing, Jiangsu, China).

### 4.2. Cell Culture

C2C12 mouse myoblast cell line was purchased from the Stem Cell Bank of the Chinese Academy of Sciences (Shanghai, China) and grown as monolayer cell cultures, as described previously [5]. Briefly, C2C12 cells were maintained in growth medium (GM), which comprised Dulbecco’s modified Eagle’s medium (DMEM; Hyclone Laboratories, Logan, UT, USA) containing 10% (*v*/*v*) fetal bovine serum (Sigma, Burlington, MA, USA), and differentiated in differentiation medium (DM), which comprised DMEM containing 2% (*v*/*v*) horse serum (Gibco, Waltham, MA, USA). Mycoplasma detection was negative. Cells were used within 5 passages after resuscitation. All cells were incubated at 37 °C in an atmosphere containing 5% CO_2_ and 95% air.

### 4.3. Animals

Ten male and ten female four-week-old ICR mice were purchased from Qinglong Shan Animal Breeding Center (Nanjing, China). After being raised for four weeks, the mice were mated. Vaginal plugs were checked on the following day. Pregnant mice were slaughtered to obtain embryos at embryo 10.5 (E10.5), embryo 12.5 (E12.5), embryo 14.5 (E14.5), embryo 16.5 (E16.5) and embryo 18.5 (E18.5); neonatal mice were slaughtered on the day of birth (B0) and 9 days (B9) after birth. For embryonic stages, the experimental unit was an entire litter, with embryos from each litter pooled for RNA extraction. For postnatal stages, the experimental unit was a single mouse. Three litters or three mice were used per time point (*n* = 3 per group). No anesthetic treatment was performed for injection experiments. All animals received no drug treatment and were in a healthy state before the experiment. Mice were raised at the Animal Husbandry Center of Yangzhou University, housed with free access to water and food at room temperature. No adverse events occurred during the processing. Mice were randomly assigned to different groups during the experiment. At the experimental endpoint, the experimental mice were euthanized by cervical dislocation. The same investigator was aware of group allocation during allocation and experiment conduct, whereas outcome assessment and data analysis were performed blinded to group allocation. All animal procedures were approved by the Animal Care and Use Committee of Yangzhou University (Approval No. SCXK-2022-0009) and performed in accordance with institutional guidelines.

### 4.4. Plasmids, RNA Oligonucleotides and Cell Transfection

Zfp36l1 siRNA (siZfp36l1), p21 siRNA (sip21), and negative control siRNA (siNC) were purchased from GenePharma (Shanghai, China); sequences are listed in Table S1. The Zfp36l1 overexpression plasmid was synthesized by Tsingke (Nanjing, China). Transfections were carried out using Lipofectamine 3000 (Thermo Fisher Scientific, Waltham, MA, USA) according to the manufacturer’s protocol.

### 4.5. RNA Extraction and Quantitative Real-Time PCR (qRT-PCR)

The method was performed as previously described [5,9]. Briefly, total RNA was extracted from cultured cells and tissues using TRIzol reagent (Invitrogen, Carlsbad, CA, USA). Messenger RNA was reverse-transcribed into cDNA using PrimeScript RT Master Mix (TaKaRa, Kusatsu, Shiga, Japan) according to the manufacturer’s instructions. qRT–PCR was performed on a Step-One Plus Real-Time PCR System using AceQ qPCR SYBR Green Master Mix (TakaRa). The relative mRNA levels were calculated by the 2^−∆∆ct^ method and normalized against those of *Gapdh*. All primers were synthesized by GENEWIZ (Suzhou, China), and their sequences are listed in Table S2. Prior to formal qRT-PCR experiments, the amplification efficiency of all primers was validated.

### 4.6. Protein Extraction and Western Blot Analysis

Total proteins were isolated from cultured cells and skeletal muscle tissues using RIPA lysis buffer supplemented with a 1% cocktail of protease and phosphatase inhibitors. Western blot analysis was carried out following our previously established protocols [9]. Antibody information is listed in Table S3.

### 4.7. Analysis of Apoptosis by Annexin V-FITC Staining

C2C12 cells at 0 and 24 h after differentiation induction, or transfected with Zfp36l1/p21 siRNAs, were harvested for apoptosis detection according to our previous publications [5,9]. Briefly, C2C12 cells were trypsinized and washed twice with PBS (HyClone, Logan, UT, USA). Then, cells were resuspended in 500 μL binding buffer, and incubated with 5 µL Annexin V-FITC and 5 µL propidium iodide (PI) for 5 min in the dark. Samples were assessed on a FACS Calibur flow cytometer (Becton Dickinson, San Diego, CA, USA). Data were analyzed using FlowJo v10.10.1 (Ashland, OR, USA). A total of 10,000 cells were analyzed per sample.

### 4.8. Cell Cycle Analysis

C2C12 cells at 0 and 24 h after differentiation induction, or transfected with Zfp36l1/p21 siRNAs were harvested for cell cycle distribution analysis according to our previous publication [9]. C2C12 cells were trypsinized, washed with phosphate-buffered saline (PBS), and fixed in precooled 75% (*v*/*v*) ethanol overnight at 4 °C. The following day, ethanol was removed after centrifugation, and cells were resuspended in 400 µL PBS and then incubated with 20 µL RNaseA solution for 30 min at 37 °C. Finally, cells were incubated with 400 µL PI staining solution for 60 min at 4 °C in the dark. The RNaseA and PI staining solutions were obtained from Cell Cycle Assay Kit (Vazyme, Nanjing, China). Samples were assessed on a FACS Calibur flow cytometer (Becton Dickinson, Franklin Lakes, NJ, USA). Data were analyzed using ModFit LT v3.2 (Verity Software House, Topsham, ME, USA). A total of 20,000 cells were analyzed per sample.

### 4.9. Immunofluorescence (IF) Assay

An immunofluorescence assay was performed to determine the γ-H2AX positive cells. Cells with obvious intranuclear γ-H2AX fluorescent foci are defined as γ-H2AX positive cells, indicating the occurrence of DNA double-strand damage. The detailed procedures were conducted as reported in our previous publications [5,9]. Briefly, cells were fixed in 4% paraformaldehyde for 1 h, permeabilized with 0.2% Triton X-100 for 10 min at 4 °C, and blocked with 5% BSA. Primary antibody against γ-H2AX (1:100 dilution; Proteintech, Rosemont, IL, USA) was applied overnight at 4 °C, followed by blocking in 5% BSA for 1 h. Next, cells were incubated for 2 h with Alexa Fluor^®^ 647 Goat Anti-Mouse IgG H&L (1:200 dilution; Abcam, Cambridge, UK). Cell nuclei were stained with Ready-to-use DAPI (KGA1808-10, KeyGEN, Nanjing, Jiangsu, China) for 15 min. Finally, mount the coverslips with mounting medium (P0128M, Beyotime, Haimen, Jiangsu, China). Fluorescent images were captured using a Zeiss LSM 710 META confocal microscope (Carl Zeiss AG, Oberkochen, Germany). Quantitative analysis of γ-H2AX-positive cells was performed using Fiji (ImageJ v1.54f).

### 4.10. RNA-Seq Analysis

C2C12 cells were collected in TRIzol reagent at 24 h post-transfection with Zfp36l1 siRNA or negative control siRNA. RNA-seq libraries were prepared and sequenced by GenoDenovo (Guangzhou, China) using an Illumina NovaSeq 6000 platform (llumina, Inc., San Diego, CA, USA). Differential expression analysis was performed using DESeq2 (v1.30.0) with thresholds of false discovery rate (FDR) < 0.05 and |fold change| ≥ 2. Gene set enrichment analysis (GSEA) was conducted using MSigDB to identify significantly enriched Gene Ontology (GO) terms, KEGG pathways, Reactome pathways, and Disease Ontology (DO) terms.

### 4.11. Comet Assay

DNA damage was assessed using a Comet Electrophoresis Kit (KeyGEN Biotech, Nanjing, China) according to the manufacturer’s instructions. Briefly, C2C12 cells were mixed with molten agarose at 37 °C and spread onto comet slides. Cells were lysed, subjected to alkaline electrophoresis (25 V, 30 min), neutralized, and stained with a DNA dye. Comet images were captured using a fluorescence microscope (Olympus, Tokyo, Japan), and the percentage of tail DNA was analyzed using CometScore v1.5 software (TriTek Solutions, Inc., Rancho Santa Margarita, CA, USA).

### 4.12. Statistical Analysis

All quantitative data were obtained from at least three independent biological replicates and are presented as mean ± SD. Comparisons between two groups used two-tailed unpaired Student’s *t*-test. For three or more groups, one-way ANOVA followed by Tukey’s post hoc test was applied. Prism 6 (GraphPad) was used for all analyses. A *p*-value < 0.05 was considered statistically significant.

## 5. Conclusions

In summary, this study identifies Zfp36l1 as a critical regulator that maintains genome stability during myogenic differentiation. Zfp36l1 is strongly induced during early myogenesis and prevents excessive DNA damage by sustaining homologous recombination repair. Mechanistically, Zfp36l1 acts via repressing p21 to preserve E2F1-dependent Rad51 transcription. Our findings reveal a previously unrecognized post-transcriptional mechanism linking RNA-binding protein function to DNA damage homeostasis and provide a molecular basis for understanding muscle development and related diseases.

## Data Availability

The original contributions presented in this study are included in the article/Supplementary Material. Further inquiries can be directed to the corresponding authors. RNA-seq data have been submitted to the China National Center for Bioinformation (CNCB) under Bioproject ID: PRJCA060050.

## Supplementary Materials

The following supporting information can be downloaded at: https://www.mdpi.com/article/10.3390/ijms27125319/s1.

## Abbreviations

AREs	AU-rich elements
CASP3	Caspase 3
cDNA	complementary DNA
DDR	DNA damage response
DEGs	Differentially expressed genes
DM	Differentiation medium
DO	Disease Ontology
DSBs	DNA double-strand breaks
FDR	False discovery rate
GM	Growth medium
GO	Gene Ontology
GSEA	Gene set enrichment analysis
HR	homologous recombination
IF	Immunofluorescence
Myh3	embryonic myosin
MyHC	myosin heavy chain
NHEJ	Non-homologous end joining
PBS	phosphate-buffered saline
PI	propidium iodide
RBPs	RNA-binding proteins
ROS	reactive oxygen species
SASP	senescence-associated secretory phenotype
SD	standard deviation
siNC	siRNA negative control
sip21	p21 siRNA
siZfp36l1	Zfp36l1 siRNA
γ-H2AX	phosphorylation of H2AX
