# Supplementary material for: Zfp36l1 Inhibits DNA Damage by Regulating p21-E2F1-Rad51 Signaling During Myogenic Differentiation
Source: Int J Mol Sci. 2026 Jun 12;27(12):5319. doi: 10.3390/ijms27125319 (PMC13300022; doi:10.3390/ijms27125319)
Supplement: Supplementary file 1 [file ijms-27-05319-s001.zip › ijms-4322032-supplementary.pdf]

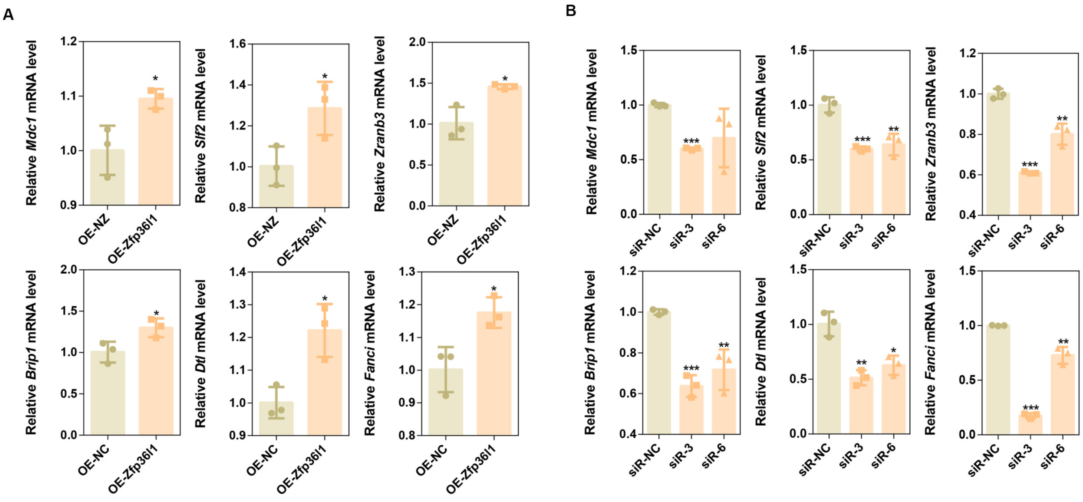

Figure S1. Zfp36l1 regulates the expression of DNA damage repair-related genes. **(A)** qRT-PCR analysis of DNA damage repair genes after Zfp36l1 overexpression. **(B)** qRT-PCR analysis of DNA damage repair genes after Zfp36l1 knockdown. Data are mean ± SD. *P < 0.05, **P < 0.01, ***P < 0.001.

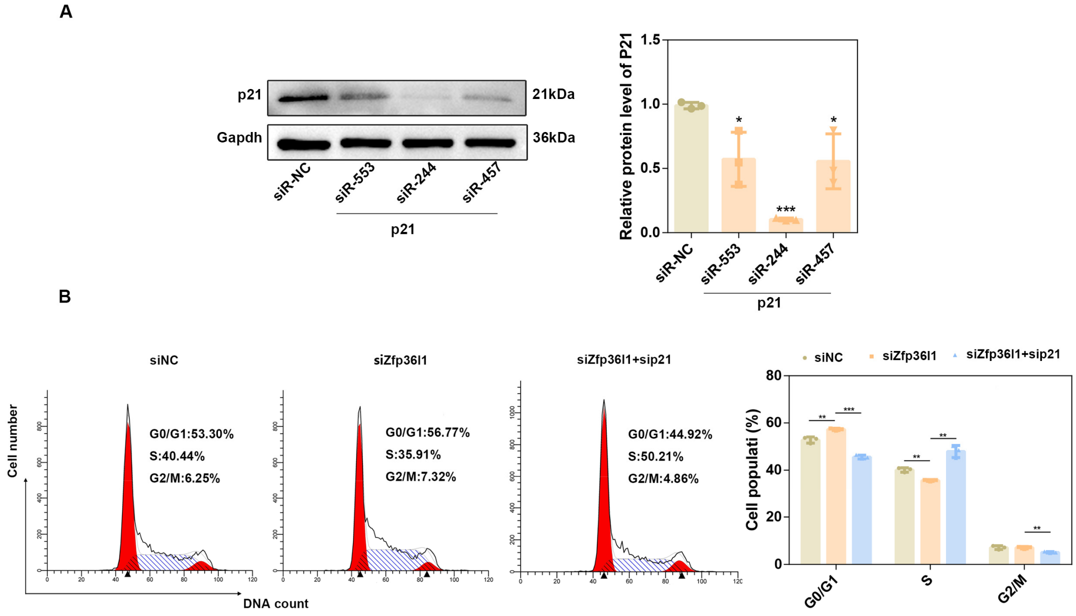

Figure S2. Knockdown of p21 rescues DNA damage induced by Zfp36l1 silencing. **(A)** Knockdown efficiency of p21 was detected by western blot analysis. GAPDH served as a loading control. **(B)** Flow cytometry analysis of cell cycle distribution showing percentages of cells in G0/G1, S, and G2/M phases in rescue experiments with co-knockdown of Zfp36l1 and p21. Data are mean ± SD. $^{*}P < 0.05$, $^{**}P < 0.01$, $^{***}P < 0.001$.

Table S1 Sequences of RNA Oligonucleotides

Name		Sequence (5'-3')
Negative control	sense:	GGAGUUCUUUGCCCGUAAUTT
	anti-sense:	AUUCGGGCAAAGAACUCCTT
Zfp36l1 siR-3	sense:	GCUCCAAGUUCCAUCAGAATT
	anti-sense:	UUCUGAUGGAACUUGGAGCTT
Zfp36l1 siR-6	sense:	GCUGCCACUUCAUUCAUAATT
	anti-sense:	UUAUGAAUGAAGUGGCAGCTT
p21 siR-553	sense:	GCAGAUUGGUCUUCUGCAATT
	anti-sense:	UUGCAGAAGACCAAUCUGCTT
p21 siR-244	sense:	GGUGGAACUUUGACUUCGUTT
	anti-sense:	ACGAAGUCAAAGUUCCACCTT
p21 siR-457	sense:	UCUGAGCGGCCUGAAGAUUTT
	anti-sense:	AAUCUUCAGGCCGCUCAGACA

Table S2 Primers sequences for qRT-PCR

Gene name		Primer sequence (5’-3’)
MyHC	F:	ACAGACATTTCCCAAATCCA
	R:	ATGTTCTTCTTCATCCGCTCC
Myh3	F:	TCCAAACCGTCTCTGCACTGTT
	R:	AGCGTACAAAGTGTGGGTGTGT
Pax3	F:	GACAGTCTGCCCACATCTCA
	R:	AGATAATGAAAGGCACTTTGTCCA
Zfp36l1	F:	AGCGAAGTTTTATGCAAGGGTAACA
	R:	CTGAGAAGCTGGTTCTGATGGA
Gapdh	F:	ATCACTGCCACCCAGAAGACT
	R:	CATGCCAGTGAGCTTCCCGTT
Cdkn1a	F:	TCCAGACATTCAGAGCCACAG
	R:	AAAGTTCCACCGTTCTCGGG
F2r	F:	ATGAGCCAGCCAGAATCAGAG
	R:	CTCCAGCAGGACGCTTTCAT
Prl2c2	F:	AGCCAGGCTCACACACTATG
	R:	TGAGGGCATTAACCCCGTTC
Tfrc	F:	CTTCGCAGGCCAGTGCTA
	R:	TGGTTCCCCACCAAACAAGT
Myzap	F:	AGGCTGATTGAACGCATGGA
	R:	CAGCTGCTGGTACCTGTTCT
Csf2rb	F:	GCAGAACTAAATGTCATGGGGC
	R:	CGGGGTTCTGTATTGGTGCT
Arhgap11a	F:	ATTAAGGTCAAGGGTGGCCGA
	R:	CGCATCAACAAGAAAGCTTGGA
Erfe	F:	TGTCCTCTATCTACAGGCAGG
	R:	TGCTTGGTAAGGGTAAGAGCC
Myb12	F:	CCTTCTCTCCTTCCCAGTTTCTG
	R:	GGGAGTACTTCTGATGATGGATAC
Cep55	F:	AGCTCCAAGTCAGACACTGC
	R:	GCTTCAAGAACTTGGATTTTCTCCA
Csn3	F:	ACTGTGGCCAATCCTGAAGC
	R:	GTTGAAATTTGGTTCCAGACCTTT
Mdc1	F:	AGTGCCAAAGAGTGCTGTGA
	R:	AACTCTGGACAGCTTCTGGG
SIf2	F:	GCTTCTCCAGCCACAGCTCA
	R:	ATGATTGACTGTTTCCTGTCCCC
Zranb3	F:	AGCAAGAAGAAGGCTCCCAC
	R:	ATCAGCCACCATACACCTGC
Brip1	F:	GATGGCAAGCACGGGAAGT
	R:	CGAGCTGTATAGTAGGGACAAGC
Dtl	F:	TCTTAGTGGCGGGAGTTGGA

	R:	TGTGAAGACCACCCGTTCC
Fanci	F:	GCCCCCACTGTCTGTTTACT
	R:	TTGGTGGAACGGCTTGAGAA
β-actin	F:	GACCTCTATGCCAACACAGT
	R:	AGTACTTGCGCTCAGGAGGA
Rad51	F:	TCCTTTACCAAGCGTCAGCC
	R:	ACTGCGACACCAAACTCATCA

Table S3 Antibody information

Name	Company	Cat. No.	Dilution
Gapdh	Proteintech	60004-1-Ig	1:5000 for WB
MyHC	DSHB	MF20	1:1000 for WB
CASP3	Proteintech	19677-1-AP	1:2000 for WB
γ-H2AX	Proteintech	83307-2-RR	1:1000 for WB
γ-H2AX	Proteintech	83307-2-RR	1:100 for IF
H2AX	Proteintech	68888-1-IG	1:1000 for WB
Zfp36l1	Proteintech	12306-1-AP	1:800 for WB
p21	Proteintech	10355-1-AP	1:1000 for WB
E2F1	ZENBIO	R24159	1:1000 for WB
Goat Anti-Rabbit IgG, HRP Conjugated	CWBIO	CW0103S	1：5000 for WB
Goat Anti-Mouse IgG, HRP Conjugated	CWBIO	CW0102S	1：5000 for WB
Goat Anti-Rabbit IgG H&L (Alexa Fluor® 488)	Abcam	ab150077	1：200 for IF
Goat Anti-Mouse IgG H&L (Alexa Fluor® 647)	Abcam	ab150115	1：200 for IF

Table S4 Differentially expressed genes between siNC and siZfp36l1 groups

**Id**	**log2(fc)**	**FDR**	**Symbol**
ncbi_12428	-2.40765908	0	Ccna2
ncbi_14062	2.22331544184503	0	F2r
ncbi_17218	-2.627192539	0	Mcm5
ncbi_17219	-2.511222413	0	Mcm6
ncbi_18140	-2.199457629	0	Uhrf1
ncbi_51788	-2.304023547	0	H2AZ1
ncbi_56338	2.82886624751362	0	Txnip
ncbi_118568032	-1.947143602	1.70948759821422e-286	gag
ncbi_66929	-2.460339605	3.02220623546159e-280	Asf1b
ncbi_13730	-1.697769024	1.64816058550884e-276	Emp1
ncbi_69065	-2.823719633	3.34327863628645e-269	Chac1
ncbi_20135	-2.012034614	5.10723241552787e-269	Rrm2
ncbi_235559	-2.371994738	3.64719782729554e-267	Topbp1
ncbi_76687	-2.539853183	3.85028004891323e-267	SPCS3
ncbi_108907	-3.025380214	3.83986885725527e-244	Nusap1
ncbi_100041194	1.52561867841671	9.83736664471784e-241	AHNAK2
ncbi_110829	-2.374316924	1.06678414651235e-239	Lims1
ncbi_26934	-1.561562121	1.28067917058398e-238	Racgap1
ncbi_17220	-1.903587288	5.65454814185849e-228	Mcm7
ncbi_218236	-1.549171386	2.19755607282787e-227	FAM120A
ncbi_14235	-1.822951415	2.74572543598582e-225	Foxm1
ncbi_13178	-3.23123183	7.54016488625534e-216	Dck
ncbi_19348	-1.660198266	9.66257519274908e-213	Kif20a
ncbi_54141	-1.997870979	3.6861733018016e-208	Spag5
ncbi_21917	-1.447651823	4.07607101714077e-206	Tmpo
ncbi_13433	-1.525503011	5.82252767341288e-206	Dnmt1
ncbi_21877	-2.5561098	1.02263598010878e-204	Tk1
ncbi_17216	-1.991233606	7.43955158583518e-200	Mcm2
ncbi_18538	-1.672427973	4.68174207845162e-199	Pcna
ncbi_102866	1.72401538005645	8.24909488303991e-198	Pls3
ncbi_17217	-1.717292444	3.94304204226509e-195	Mcm4
ncbi_97165	-1.744428883	5.90984317465564e-192	Hmgb2
ncbi_20620	1.75436217294008	7.48718958076164e-192	Plk2
ncbi_51944	-2.072705749	2.24212353435477e-189	Knstrn
ncbi_233406	-1.614704673	1.43725801388858e-184	Prc1
ncbi_12534	-1.897866165	5.00370341350476e-182	Cdk1
ncbi_68275	-1.522282483	7.86255693743433e-182	Rpa1
ncbi_16319	-1.80855033	1.16218284109865e-179	Incenp
ncbi_20133	-1.635454009	4.26194134005684e-177	Rrm1
ncbi_16647	-1.327569545	1.06400846657452e-176	Kpna2
ncbi_270906	-1.935878253	2.53772138684165e-176	Prr11
ncbi_72119	-1.548472581	1.18981910059871e-175	Tpx2

ncbi_105988	-1.822467044	1.63369242522097e-173	Espl1
ncbi_11752	2.32750608250212	1.31102907294893e-169	Anxa8
ncbi_68743	-1.741988075	1.04095765026417e-168	Anln
ncbi_16881	-1.942181108	9.08014282719697e-168	Lig1
ncbi_18000	-1.277348809	9.38789767931432e-168	Septin2
ncbi_20085	-2.827360173	3.03223722796809e-158	Rps19
ncbi_18973	-1.87060416	2.24254322047373e-154	Pole
ncbi_67951	-1.441825945	4.82982486210988e-154	Tubb6
ncbi_12236	-1.601551902	4.59692967840993e-153	Bub1b
ncbi_381280	-1.568577188	3.20022180126842e-152	Hjurp
ncbi_11911	-1.287176986	3.82601034702088e-152	Atf4
ncbi_16997	1.36968891144852	1.96503682816699e-151	Ltbp2
ncbi_17215	-1.687761091	4.42097423171681e-150	Mcm3
ncbi_97064	-1.577166329	1.51590421177266e-149	Wwtr1
ncbi_268697	-1.733214884	2.62176309906467e-149	Ccnb1
ncbi_18811	1.66061515290036	9.19196237885504e-146	Prl2c2
ncbi_107823	-1.662984599	4.20043652404685e-145	Nsd2
ncbi_14793	-1.859306477	9.12293890504037e-145	Cdca3
ncbi_68298	-1.426562485	9.52490385106287e-145	Ncapd2
ncbi_12340	-1.2952818	5.19214484346578e-144	Capza1
ncbi_12387	-1.109932104	7.1767563789461e-144	Ctnnb1
ncbi_18817	-1.86963757	2.0647328782517e-143	Plk1
ncbi_404710	-1.833574237	3.11992961231427e-143	IQGAP3
ncbi_21973	-1.704435288	5.2644402080174e-142	Top2a
ncbi_17222	-1.172359429	6.26094266355325e-142	Anapc1
ncbi_230484	-1.443621292	1.14493841864522e-141	Usp1
ncbi_18787	1.42796043492771	2.45202361707912e-140	Serpine1
ncbi_17279	-1.909709318	1.03707253646504e-136	Melk
ncbi_170676	-1.237646562	1.25846609021451e-135	Peg10
ncbi_14489	-1.177745082	1.8007740388787e-134	Mtpn
ncbi_236792	-2.01377285	2.65577360749072e-133	Mmgt1
ncbi_22042	1.99619917865144	6.30529933121839e-133	Tfrc
ncbi_12442	-1.689701387	9.19544710444576e-133	Ccnb2
ncbi_27494	-1.664326747	1.69568663280555e-132	Amot
ncbi_68612	-1.557458595	4.64976170094531e-131	Ube2c
ncbi_110033	-1.797176512	1.05762457037038e-130	Kif22
ncbi_98238	-1.237533457	2.31864567712496e-130	Lrrc59
ncbi_12389	-1.057912964	9.06509301350726e-130	Cav1
ncbi_215387	-1.750654058	9.36166345229277e-130	Ncaph
ncbi_27367	-1.112209961	1.9776712942564e-129	Rpl3
ncbi_105245097	-2.201950239	2.81341609943872e-128	gag-pol
ncbi_22628	1.27228572371569	3.61998293579539e-128	YWHAG
ncbi_52276	-1.928669585	1.18060402429869e-126	Cdca8
ncbi_227613	-1.098170234	3.79151131329908e-126	TUBB4B

ncbi_21335	-1.690648021	1.28297230915734e-125	Tacc3
ncbi_16765	-1.561979475	5.30662983501069e-125	Stmn1
ncbi_21351	1.96849131269074	7.3384258895562e-125	Taldo1
ncbi_20877	-2.147304482	4.90832128511743e-124	Aurkb
ncbi_12338	-1.359387403	7.68568016708412e-124	Capn6
ncbi_56150	-1.821400398	1.11834540701022e-122	Mad2l1
ncbi_106795	-2.481339073	8.18435957965487e-122	Tcf19
ncbi_68842	1.14614564766373	1.07344283980052e-121	Tulp4
ncbi_12400	-1.552993779	1.38032648847197e-121	Cbfb
ncbi_26362	-1.129851031	1.97090228987638e-120	Axl
ncbi_14672	-1.418528324	1.97657772152319e-119	Gna11
ncbi_52033	-2.116163021	5.09833839962698e-119	Pbk
ncbi_70218	-1.795906009	1.28397258355289e-118	Kif18b
ncbi_108167918	-1.834486228	7.95458863338826e-118	env
ncbi_11736	-1.278971086	9.18704944242459e-118	Ankfy1
ncbi_12189	-2.050626073	2.14499570731093e-116	Brca1
ncbi_20460	-2.254807226	4.7284482167818e-116	Stil
ncbi_107435	-1.922504273	1.42363242953591e-115	Hat1
ncbi_17865	-2.347191345	2.81336988662008e-115	Mybl2
ncbi_17698	-1.029721409	6.63898897737911e-115	Msn
ncbi_13605	-1.504430048	8.186027320212e-115	Ect2
ncbi_107995	-1.405136873	1.47281117600783e-114	Cdc20
ncbi_11799	-1.728331511	1.93879724217454e-114	Birc5
ncbi_22319	-1.335394377	3.36039997091697e-114	Vamp3
ncbi_12449	-1.702096135	1.75480069993008e-113	Ccnf
ncbi_67177	-2.278665077	4.06902755971338e-113	Cdt1
ncbi_12519	1.35631585491889	6.32829415911341e-112	Cd80
ncbi_68026	-2.027930574	1.04678344147716e-111	Pclaf
ncbi_16551	-1.742786236	2.07191010785366e-110	Kif11
ncbi_12235	-2.04326123	1.25719450386725e-109	Bub1
ncbi_66471	-1.189685673	2.63910918701998e-109	Anp32e
ncbi_14860	1.34150031694861	3.4626265637491e-109	Gsta4
ncbi_73804	-1.591067147	7.62231353332655e-108	Kif2c
ncbi_17988	-1.227664546	2.54050006986183e-106	Ndrg1
ncbi_74107	-1.870233898	7.42439942291963e-105	Cep55
ncbi_108912	-1.67981165	1.02083932462727e-104	Cdca2
ncbi_234593	1.45895813381201	5.4637922413175e-104	ndrg4
ncbi_11615	-1.146060955	2.09148408338309e-103	Ahcy
ncbi_108671	-2.016547004	2.93521761760534e-103	Dnajc9
ncbi_240087	-1.473341222	3.29070003184918e-103	Mdc1
ncbi_66442	-2.055698146	7.1337473844699e-103	Spc25
ncbi_56334	-1.019689476	1.91361424320495e-102	Tmed2
ncbi_56495	-1.244910042	2.48947415525396e-102	Get3
ncbi_21853	-2.291180595	6.03524051274751e-101	Timeless

ncbi_70466	-1.691388886	7.52097908223651e-101	Ckap2l
ncbi_67819	-1.389459758	4.56515833233289e-100	Derl1
ncbi_64209	-1.353206907	7.75205155706434e-100	Herpud1
ncbi_227059	-2.047576722	9.27979857775411e-100	Slc39a10
ncbi_17768	-1.410007595	1.52551627048736e-99	Mthfd2
ncbi_66676	-1.47956749	5.92845307814306e-99	TMED7
ncbi_71819	-1.475818638	1.50110911479163e-98	Kif23
ncbi_72415	-2.112413462	7.12030108655338e-98	Sgo1
ncbi_69227	-1.633624365	1.04988882526346e-97	-
ncbi_11670	1.78164848757806	1.5757142125711e-97	Aldh3a1
ncbi_58207	-1.835374456	2.14023268100303e-97	Slc43a3
ncbi_22057	1.53573019210037	2.19318957847727e-97	Tob1
ncbi_228482	-1.569659218	7.63509425614565e-97	Arhgap11a
ncbi_19650	-1.598228523	8.70762783295373e-97	Rbl1
ncbi_12575	1.13840095011411	1.99599505403158e-96	Cdkn1a
ncbi_237436	-1.876574647	2.00319040330637e-96	Gas2l3
ncbi_67276	-1.733114757	2.28723747041217e-96	Eri1
ncbi_16906	-1.370706632	4.47612563749391e-95	Lmnb1
ncbi_54208	-1.483438579	5.23794307968459e-95	Arl6ip1
ncbi_98878	-1.366498848	5.2893846301012e-95	Ehd4
ncbi_20937	-1.606170395	2.83341747357627e-94	Suv39h1
ncbi_118568312	2.58088337533455	6.70524239345517e-93	--
ncbi_13836	1.25288249153156	1.37173627764518e-92	Epha2
ncbi_227358	-2.379947383	2.53991428786803e-92	Erfe
ncbi_228775	-2.1371462	4.63925485052992e-92	Trib3
ncbi_56473	-1.015177075	1.67405020686668e-89	Fads2
ncbi_118568792	-1.984613856	3.38974285700285e-89	pol
ncbi_66427	-1.048764191	3.79604612624405e-89	Cyb5b
ncbi_19384	-1.018669948	8.18184548291404e-89	RAN
ncbi_60530	-1.925366895	2.01599373663563e-88	Fignl1
ncbi_108037	-1.059253507	1.14673748670196e-87	Shmt2
ncbi_11671	-1.390876839	2.02151284136052e-87	Aldh3a2
ncbi_225363	1.04111446782887	2.72708331541871e-87	ETF1
ncbi_236930	-1.803269893	3.24172617325582e-87	Ercc6l
ncbi_52679	-1.650107072	5.3584223033716e-87	E2f7
ncbi_27221	-1.74773465	1.69201643936008e-86	Chaf1a
ncbi_72007	1.08902108709395	1.72734628784333e-86	Fndc3b
ncbi_237911	-2.492882951	2.90512110019709e-86	Brip1
ncbi_18176	-1.138228389	4.54681755888819e-86	NRAS
ncbi_381293	-1.927684127	7.66488463137958e-86	Kif14
ncbi_19230	-1.245518263	1.81358872100449e-85	Twf1
ncbi_56445	-1.015332039	1.85598802575873e-85	Dnaja2
ncbi_11787	1.25868495438336	1.90875580449082e-85	Apbb2
ncbi_116972	-1.777674826	2.27718551758653e-85	Tlcd3a

ncbi_269582	-1.899295028	4.20847697506526e-85	Clspn
ncbi_100502766	-1.936553125	4.25369128723296e-85	Kifc1
ncbi_65112	1.43700168108072	9.5730752369461e-84	Pmepa1
ncbi_14056	-1.430571715	3.57003247973136e-83	Ezh2
ncbi_18005	-1.442955258	2.23149988269435e-82	Nek2
ncbi_12316	-1.68552531	5.62839890964512e-82	Aspm
ncbi_14182	1.09085431897454	6.29486657432741e-82	Fgfr1
ncbi_29864	-1.364046042	9.26549770620599e-82	Rnf11
ncbi_236539	-1.031342626	1.42930866021997e-81	Phgdh
ncbi_109212	-1.695603276	1.68894240593266e-81	Pimreg
ncbi_12632	-1.240394787	2.25938322971493e-81	Cfl2
ncbi_58184	-1.269832564	2.68429182253772e-81	CNOT9
ncbi_218977	-1.633981687	7.45049347800035e-81	Dlgap5
ncbi_67629	-2.227039075	1.5495084266641e-80	Spc24
ncbi_226178	1.35954620468622	1.66003947455776e-80	Wbp11
ncbi_216188	-1.118893323	1.61500542813572e-79	Aldh1l2
ncbi_56419	-1.309498571	1.85954261951832e-78	Diaph3
ncbi_14857	2.02354922758829	2.43542871118222e-78	Gsta1
ncbi_57875	1.68540742679808	4.35347271424255e-78	Angptl4
ncbi_12021	-1.953292049	2.1911316455066e-77	Bard1
ncbi_12521	-1.130945227	2.03603215801237e-76	Cd82
ncbi_66977	-1.875730544	5.51050530273478e-76	Nuf2
ncbi_12822	1.00327443323587	6.64243287224835e-76	Col18a1
ncbi_75605	1.79427315069624	2.10300461846589e-75	Kdm5b
ncbi_30960	-1.273476858	8.06289068406189e-75	Vapa
ncbi_54392	-1.64026428	8.21349371873964e-75	NCAPG
ncbi_73130	-1.483971647	9.39415145920915e-75	Tmed5
ncbi_67141	-2.100044968	3.71406311920559e-74	Fbxo5
ncbi_13642	1.51422381807534	3.80478664423749e-74	Efnb2
ncbi_54124	-1.425440824	1.25747014877291e-73	CKS1B
ncbi_68477	-1.697787004	3.13216596721514e-73	RMND5A
ncbi_12704	-1.141006923	3.60273118795855e-73	CIT
ncbi_74551	-1.413916608	5.06090552999985e-73	Pck2
ncbi_74016	-1.854534624	7.17751895712739e-73	Phf19
ncbi_19366	-2.468093179	8.37857555089702e-73	Rad54l
ncbi_118568683	-1.07032101	2.63791318978117e-72	env
ncbi_445007	-1.56997535	4.45519205560397e-72	Nup85
ncbi_16571	-1.585418996	4.74269288696777e-72	Kif4
ncbi_22152	1.1906626936627	5.83274741789917e-72	Tubb3
ncbi_20454	-1.341261846	9.35976105723712e-72	St3gal5
ncbi_79456	-2.169523548	1.87214884076136e-71	Recql4
ncbi_11636	1.13074842929772	3.45247019862894e-71	Ak1
ncbi_18792	1.15470117960916	3.84793946948931e-71	Plau
ncbi_19361	-2.294497413	4.31464121655363e-71	Rad51

ncbi_67398	-1.010081457	1.07171369327894e-70	Srpra
ncbi_69608	-1.035184944	1.78070088613606e-70	SEC24D
ncbi_117146	1.17195190437632	5.55614380973499e-70	Ube3b
ncbi_208628	-1.577436275	5.66797881163552e-70	Kntc1
ncbi_102595	1.64086790070767	7.19335575494591e-70	Plekho2
ncbi_65960	-1.118347736	7.2553407653486e-70	Twsg1
ncbi_18582	-1.583528685	1.25071781653009e-69	Pde6d
ncbi_15201	-1.721694553	1.31162218457882e-69	Hells
ncbi_77559	-1.220526867	1.7304208989285e-69	AGL
ncbi_209737	-1.688305727	2.66515704100402e-69	Kif15
ncbi_12649	-2.549620474	3.18613214931621e-69	Chek1
ncbi_76895	-1.027995473	4.62299206360881e-69	Bicd2
ncbi_15331	-1.183717758	8.07229688487289e-69	Hmgn2
ncbi_23834	-2.142922464	1.10145724288657e-68	Cdc6
ncbi_20539	-1.239965266	4.81250067928042e-68	Slc7a5
ncbi_22171	-1.539391811	7.41466516354748e-68	Tyms
ncbi_11898	1.32229402491618	8.22199157315934e-68	Ass1
ncbi_14376	1.04776585198861	3.474536963051e-67	Ganab
ncbi_70024	-2.023041122	8.71280444689016e-67	Mcm10
ncbi_56468	1.18877000464745	9.42390867314587e-67	Socs5
ncbi_217830	1.26249608678244	2.37037537661214e-66	Dglucy
ncbi_77011	-2.098642014	3.68390894873008e-66	Ticrr
ncbi_108682	-1.175078539	4.41239450678198e-66	Gpt2
ncbi_15511	1.47844431518562	7.6357101890149e-66	Hspa1b
ncbi_212377	-2.008795293	1.04384555674698e-65	Mms22l
ncbi_54214	1.34605786685379	1.25364212975778e-65	Golga4
ncbi_30878	-1.919708983	1.30613306191559e-65	Apln
ncbi_69263	-1.674635221	3.57638930525593e-65	Rfc3
ncbi_12580	-1.447092087	8.86721865831746e-65	Cdkn2c
ncbi_106618	-1.868091145	2.63287448454205e-64	Wdr90
ncbi_217558	-1.453292031	4.33109066482619e-64	G2e3
ncbi_78658	-1.272519754	4.33109066482619e-64	Ncapd3
ncbi_210544	-1.369483468	4.80353397444052e-64	Tbc1d31
ncbi_56048	-1.204475226	8.66996135963195e-64	Lgals8
ncbi_272551	-3.17131415	9.8581316261561e-64	Gins2
ncbi_234258	-2.028254511	1.08110560351423e-63	Neil3
ncbi_276846	1.30919423790618	1.21362719288852e-63	Pigs
ncbi_224088	-1.127099553	3.68779473363712e-63	Atp13a3
ncbi_20257	1.07074721604107	4.19311938590344e-63	STMN2
ncbi_20878	-1.343224851	4.21291463134771e-63	Aurka
ncbi_19242	-1.528095605	3.16628008790898e-62	Ptn
ncbi_382030	-1.257757998	7.25089494431759e-62	CNEP1R1
ncbi_16728	1.26483237624213	7.27823260323362e-62	L1cam
ncbi_13655	2.31081241734012	1.02257934659824e-61	Egr3

ncbi_102920	-1.632654706	1.35400888924589e-61	Cenpi
ncbi_105837	-1.241443893	1.8590653144924e-61	Mtbp
ncbi_67553	-1.857151045	2.13888632944626e-61	Gstcd
ncbi_140484	-1.455027368	2.43803944507893e-61	Pofut1
ncbi_18971	-1.508141289	3.45380206946251e-61	Pold1
ncbi_19934	-1.351700157	8.49472539281673e-61	RPL22
ncbi_30939	-1.546461499	1.00117784329186e-60	Pttg1
ncbi_12424	2.64172821183333	1.59377820490278e-60	Cck
ncbi_18968	-1.676244953	3.50164500866201e-60	Pola1
ncbi_218973	-1.590462062	5.75318487365627e-60	Wdhd1
ncbi_18969	-1.479690851	6.21182343292768e-60	Pola2
ncbi_66197	-1.682224978	8.905818200963e-60	Cks2
ncbi_218442	-1.509246965	1.03408719986753e-59	Serinc5
ncbi_19108	1.35160686583557	1.34707824762891e-59	Prkx
ncbi_20315	-1.402640173	1.75600865020191e-59	Cxcl12
ncbi_13361	-1.817586948	2.1359892483199e-59	Dhfr
ncbi_50790	-1.475444394	2.91442887040042e-59	Acsl4
ncbi_12192	-1.626124172	3.90829097100903e-59	Zfp36l1
ncbi_16164	-1.317239578	6.67800142339551e-59	Il13ra1
ncbi_14156	-1.75370742	1.06864002737304e-58	Fen1
ncbi_11303	2.40172044486851	1.58305907761157e-58	Abca1
ncbi_15366	-1.328805138	3.82091989658401e-58	Hmmr
ncbi_16580	-1.908705336	9.69294157394514e-58	Kifc1
ncbi_231464	-1.267334822	1.38565530120168e-57	Cnot6l
ncbi_71085	-1.762285406	1.65886531885663e-57	Arhgap19
ncbi_110460	-1.148753892	3.80874808823578e-57	Acat2
ncbi_26385	-1.237405585	5.41731039157171e-57	Grk6
ncbi_17688	-1.245651943	8.99762816206699e-57	Msh6
ncbi_320910	2.82016420255616	1.15583018561651e-56	Itgb8
ncbi_20419	-1.50994238	1.75651500467155e-56	Shcbp1
ncbi_72151	-1.630077554	1.80007558188681e-56	Rfc5
ncbi_106582	-1.642914543	2.3483629265141e-56	Nrm
ncbi_11839	2.06434462239781	6.43705387805443e-56	Areg
ncbi_110639	-1.525136695	1.0884815963344e-55	Prps2
ncbi_15528	-1.598764535	1.85177537173021e-55	Hspe1
ncbi_70827	1.04323806039899	3.68985787366755e-55	Trak2
ncbi_56424	-1.098421055	3.68985787366755e-55	Stub1
ncbi_98386	-1.04886929	4.43050432044676e-55	Lbr
ncbi_110749	-1.693214038	5.39050670646184e-55	Chaf1b
ncbi_78733	-1.610123114	5.80302388059131e-55	Troap
ncbi_22137	-1.673859242	6.34261544819252e-55	Ttk
ncbi_22367	-1.630227713	6.99518375071048e-55	Vrk1
ncbi_16646	-1.122765193	9.71261418889775e-55	Kpna1
ncbi_66648	-1.194436377	1.08587822450185e-54	Tpgs2

ncbi_20444	1.21357964469242	2.5217090736358e-54	St3gal2
ncbi_75317	-1.644677644	2.86725401236186e-54	Parpbp
ncbi_268930	-2.236335404	3.40616199236681e-54	Pkmyt1
ncbi_15368	1.05128704174552	2.25352409662542e-53	Hmox1
ncbi_76843	-1.720599177	3.25684477132208e-53	Dtl
ncbi_381306	-1.912904426	3.46601104705562e-53	C1orf112
ncbi_19087	-1.215056612	4.59383206999749e-53	Prkar2a
ncbi_56336	1.01918989259947	4.90493193289446e-53	B4galt5
ncbi_66713	1.05486340666649	6.99008327329307e-53	ACTR2
ncbi_12447	-1.759940229	9.03498553908496e-53	Ccne1
ncbi_19053	-1.319807128	9.0633355001309e-53	PPP2CB
ncbi_12350	-1.212692614	2.07014216429526e-52	Ca3
ncbi_14219	1.90307357214761	2.9215221183238e-52	Ccn2
ncbi_83796	-1.147288274	6.36675584184283e-52	SMARCD2
ncbi_67037	-1.491270755	7.02581733464731e-52	Pmf1
ncbi_67414	1.24895536252855	9.61142055906426e-52	Mfn1
ncbi_65972	-1.886309651	1.09844964731808e-51	Ifi30
ncbi_76178	-1.099557907	1.42960341023756e-51	Coa5
ncbi_67052	-1.646097576	1.45818747562967e-51	Ndc80
ncbi_15270	-1.255849521	1.55898195458656e-51	H2ax
ncbi_118567918	-1.601767371	1.78301317125843e-51	env
ncbi_56045	-1.278880191	1.90986553462369e-51	Samhd1
ncbi_72749	-1.628039556	7.01344865370661e-51	Tonsl
ncbi_12904	1.30748001118221	1.21606252034309e-50	Crabp2
ncbi_29870	-1.098487294	1.44117983271448e-50	Gtse1
ncbi_12532	-1.729870806	4.12731550165401e-50	Cdc25c
ncbi_78586	-1.862879618	9.7837947048162e-50	Srbd1
ncbi_20492	-1.098612188	1.26075393103371e-49	Slbp
ncbi_12448	-1.958382033	3.26959479312316e-49	Ccne2
ncbi_16950	1.01174700063401	3.83025846259446e-49	Loxl3
ncbi_99167	-1.393128971	6.08297402031221e-49	Ssx2ip
ncbi_210711	-1.152310017	6.14691842430749e-49	Mcmbp
ncbi_24083	-1.59206974	7.29578774135592e-49	Natd1
ncbi_212168	1.15391432962939	2.15075285554555e-48	Zswim4
ncbi_18972	-1.187320008	2.53722418334957e-48	Pold2
ncbi_68201	-1.748266028	3.15506710895835e-48	Ccdc34
ncbi_27376	-1.196345189	6.23730968666876e-48	Slc25a10
ncbi_228033	-1.121739831	7.55716108590254e-48	Atp5mc3
ncbi_20657	1.01017663080327	1.10070637913446e-47	Sod3
ncbi_21847	1.0392902432079	1.32395314209701e-47	Klf10
ncbi_66468	-1.931957026	1.83891352517912e-47	Ska1
ncbi_259302	1.10938004140829	1.89525973271827e-47	Srgap3
ncbi_219114	-1.6780871	1.99339126011083e-47	Ska3
ncbi_270066	-1.046393331	2.56821213676503e-47	Slc35e1

ncbi_14904	-1.072415935	3.69245496193806e-47	Gtpbp1
ncbi_54216	1.01703672437536	9.42240179301508e-47	PCDH7
ncbi_56722	-1.042815631	1.24479192242634e-46	Litaf
ncbi_245828	-1.341835628	1.38237279859454e-46	Trappc1
ncbi_12544	-1.644849618	1.87864553284335e-46	Cdc45
ncbi_57441	-2.10584158	3.10588699508128e-46	Gmnn
ncbi_107869	-2.640695756	3.3992461522041e-46	Cth
ncbi_19891	-1.952937583	5.25727117149396e-46	Rpa2
ncbi_12615	-1.363732917	6.91936421328485e-46	Cenpa
ncbi_21871	1.07909949287179	6.93440478360724e-46	Atp6v0a2
ncbi_19362	-2.242844979	7.08638047792218e-46	Rad51ap1
ncbi_105787	1.05470131841373	9.38508704597577e-46	Prkaa1
ncbi_15200	1.71164900215263	1.60455353917475e-45	Hbegf
ncbi_75599	1.10517436582212	1.62650646876102e-45	PCDH1
ncbi_269959	-1.316279753	2.16056453004596e-45	ADAMTSL3
ncbi_74365	-1.565356017	2.16909008909595e-45	Lonrf3
ncbi_12190	-1.607324056	2.59373728464455e-45	Brca2
ncbi_19075	-1.59348883	3.43708955031264e-45	Prim1
ncbi_80905	-1.294280773	3.50419590079112e-45	Polh
ncbi_20973	-1.789844478	4.1981520332866e-45	Syngr2
ncbi_69071	-1.180187738	4.33454440884008e-45	Tmem97
ncbi_14114	-1.029737133	5.57243452397274e-45	Fbln1
ncbi_66570	-2.074405552	1.00413226662575e-44	Cenpm
ncbi_83397	1.01227766138613	1.46262431280991e-44	Akap12
ncbi_70568	-1.176120205	1.54268625333765e-44	Cpne3
ncbi_68014	-1.322228914	3.58816389283565e-44	Zwilch
ncbi_22393	-1.227599113	3.65150319976238e-44	Wfs1
ncbi_56452	-1.04279908	4.27168624769392e-44	Orc6
ncbi_70420	-1.19342101	4.27819538660662e-44	Arpin
ncbi_17997	-1.228401781	5.80481510585034e-44	Nedd1
ncbi_19076	-1.297346314	6.03641430158301e-44	Prim2
ncbi_381318	-2.353901151	6.35177701877049e-44	Nsl1
ncbi_14081	-1.361169726	9.08867865184478e-44	Acsl1
ncbi_330695	2.91058447019482	1.02852692732442e-43	Ctxn1
ncbi_53893	-1.299985716	1.67951610291923e-43	Nudt5
ncbi_11761	1.54317750884882	1.74776017272699e-43	Aox1
ncbi_77782	-1.752915929	2.84830891629075e-43	Polq
ncbi_24075	-1.514171564	3.62183744132342e-43	Taf10
ncbi_28113	-1.508780736	1.07118563010515e-42	Tinf2
ncbi_110842	-1.148644081	1.30184781282959e-42	Etfa
ncbi_77951	-1.623747438	1.53602167434033e-42	Cyp20a1
ncbi_109242	-1.48112669	1.94789568562474e-42	Kif24
ncbi_238673	-1.322585794	2.69275700629243e-42	Znf367
ncbi_208440	1.10635976678177	7.46006911317523e-42	DIP2C

ncbi_224250	-1.069953193	7.55001037571585e-42	Cldnd1
ncbi_72543	-1.691174931	1.32266199334338e-41	Mvb12b
ncbi_106344	-1.779810714	1.57916050413686e-41	Rfc4
ncbi_17207	1.47997296984334	2.03494416195719e-41	Mcf2l
ncbi_110074	-1.731625762	1.32925275050523e-40	Dut
ncbi_72787	-1.037107581	1.47399441216765e-40	Ndc1
ncbi_218581	-2.109757331	1.47496943548368e-40	Depdc1b
ncbi_76131	-1.486566598	1.98688849182009e-40	Depdc1a
ncbi_98733	-1.575600801	3.60709379181681e-40	Obsl1
ncbi_55982	-1.057153347	5.33828886253533e-40	Paxip1
ncbi_67967	-1.095732913	6.74498168040217e-40	Pold3
ncbi_170799	-1.77600291	1.15505174446644e-39	Rtkn2
ncbi_237886	-2.005722495	2.93918913833485e-39	Slfn9
ncbi_58520	-1.49813966	3.01627769633566e-39	Erg28
ncbi_60599	1.17075029554611	3.04014695194852e-39	Trp53inp1
ncbi_72155	-1.651304719	3.12358863362343e-39	Cenpn
ncbi_59056	-1.053584286	3.91602044699459e-39	Evc
ncbi_66980	-1.004848022	4.68026567698456e-39	Zdhhc6
ncbi_14583	-1.005038489	4.88886872529699e-39	Gfpt1
ncbi_14841	-1.916455576	5.94055915786021e-39	Haspin
ncbi_74412	-1.496997713	8.19729650231773e-39	Gle1
ncbi_67121	-1.352317154	1.02051285765186e-38	Mastl
ncbi_74237	-1.002891561	2.46115239196206e-38	Tubgcp2
ncbi_17121	-1.993248646	3.22277631378309e-38	Mxd3
ncbi_56312	-1.28908968	3.48815389033877e-38	Nupr1
ncbi_68549	-1.3173249	5.06910501844525e-38	Sgo2
ncbi_20747	1.1289147498143	9.3900238266946e-38	SPOP
ncbi_12778	1.0027064171536	1.06143708512156e-37	Ackr3
ncbi_237877	-1.71532285	1.65415315584085e-37	Atad5
ncbi_15499	-1.06492062	2.54003832205129e-37	Hsf1
ncbi_71988	-1.898674719	2.70643096791737e-37	Esco2
ncbi_22247	-1.052353639	3.1857353641972e-37	Umps
ncbi_72549	-1.065565171	3.26210164985178e-37	Reep4
ncbi_56471	1.90075437922013	3.63098571603893e-37	Stmn4
ncbi_12452	-1.185607603	5.88798400679507e-37	Ccng2
ncbi_192652	1.04524501589795	6.97696311461002e-37	Wdr81
ncbi_93790	-1.406982202	7.42693900034023e-37	Nipa2
ncbi_70385	-1.575608505	7.6020163287385e-37	Spdl1
ncbi_26420	-1.03937462	1.82148921523818e-36	Mapk9
ncbi_67397	-1.320029789	2.56292694214485e-36	Erp29
ncbi_67117	1.1200885862411	3.57563056650405e-36	Dynlt3
ncbi_211651	-1.534964711	5.46152896129046e-36	Fancd2
ncbi_666704	-1.082234124	1.09668770962479e-35	Samd1
ncbi_67180	-1.089590615	1.13109637626075e-35	Yipf5

ncbi_230649	-1.749628992	1.19253781462241e-35	Atpaf1
ncbi_69605	-1.194878929	2.05692252443848e-35	Lnpk
ncbi_20873	-1.304923341	2.5828490372464e-35	Plk4
ncbi_20822	1.01093066722881	2.81481267441619e-35	RO60
ncbi_235627	1.30236111787846	2.83916169671328e-35	Nbeal2
ncbi_242466	1.07391337237939	4.81392051486544e-35	Znf462
ncbi_71878	-1.235247954	1.0130924747494e-34	Fam83d
ncbi_224171	-1.182762807	1.1422230975911e-34	Cip2a
ncbi_13136	1.32344599319101	1.31933477311937e-34	Cd55
ncbi_17864	-1.721582882	1.58428835239474e-34	Mybl1
ncbi_12983	2.08269102914202	3.3884497235348e-34	Csf2rb
ncbi_67731	1.56336735309056	4.39962693843276e-34	Fbxo32
ncbi_50496	-1.247274517	6.03177111560969e-34	E2f6
ncbi_22701	-1.323384411	8.37886634090107e-34	Zfp41
ncbi_19718	-1.071355117	1.73348032020059e-33	Rfc2
ncbi_319675	-1.05399961	2.15956689724804e-33	Cep295
ncbi_72140	-1.381251158	2.23128539933678e-33	Cep89
ncbi_12033	-1.549263973	5.13457640774864e-33	Bcap29
ncbi_226151	-1.126687202	5.62486574347775e-33	Slf2
ncbi_12581	-1.494085688	9.04060997728234e-33	Cdkn2d
ncbi_226518	1.12753810792395	1.29048494560732e-32	Nmnat2
ncbi_217734	-1.176704987	1.53045195157256e-32	Pomt2
ncbi_76142	-1.526811077	1.95252890996483e-32	Ppp1r14c
ncbi_83815	-1.521952703	3.15544358124289e-32	Cenpq
ncbi_56708	1.14093172635648	3.73584203453262e-32	Clcf1
ncbi_269224	-1.506031096	3.74880484323698e-32	Pask
ncbi_19225	1.1740574640754	4.34662545265389e-32	Ptgs2
ncbi_228421	-1.622801129	4.67145939029162e-32	Kif18a
ncbi_244152	1.37548864373925	5.0530074338689e-32	Tsku
ncbi_105244833	3.60317399022097	5.72874206662696e-32	--
ncbi_217216	-1.670937126	1.593358915797e-31	Hrob
ncbi_217653	-1.482564937	2.03430049100785e-31	Mis18bp1
ncbi_208836	-1.470868681	2.43283348310583e-31	Fanci
ncbi_14087	-1.779971498	2.53929260874235e-31	Fanca
ncbi_69724	-1.347488597	2.60466769270801e-31	Rnaseh2a
ncbi_269400	-1.019009611	2.61759791308476e-31	Rtel1
ncbi_80517	-1.101858164	3.61232324130268e-31	Herpud2
ncbi_70472	-1.840803666	4.14151314165453e-31	ATAD2
ncbi_15958	-1.244207691	6.46494283930339e-31	Ifit2
ncbi_627375	-1.419817079	9.72187231032527e-31	Hmgn2
ncbi_234967	-1.354395814	1.00254767163194e-30	Slc36a4
ncbi_105513	-1.21936158	1.73376660889843e-30	Chmp7
ncbi_12144	-1.655282851	2.05622182323496e-30	Blm
ncbi_74044	-1.25176958	4.11127979181664e-30	Ttf2

ncbi_66711	1.06810008207222	4.64388655151588e-30	Sbds
ncbi_232086	1.6767414091369	7.68668300167711e-30	Tmem150a
ncbi_67246	1.01605311938354	8.20434166796403e-30	Resf1
ncbi_18767	-1.125920528	9.54742469955169e-30	Pkia
ncbi_67849	-1.722221422	9.96433677606715e-30	Cdca5
ncbi_57435	1.36220090539132	1.101877251496e-29	Plin4
ncbi_108767	1.04782344914631	1.32765743438759e-29	Pnrc1
ncbi_230784	-1.02475155	1.33111518771182e-29	Sesn2
ncbi_21844	1.09200936999515	2.89341087023849e-29	Tiam1
ncbi_269855	1.07477191562663	2.9169809505212e-29	Ssc5d
ncbi_71909	-1.752250485	3.38651001514992e-29	Haus5
ncbi_94219	1.55743469133167	3.94697274114872e-29	Cnnm2
ncbi_66645	-1.027794747	5.14555869323866e-29	Pspc1
ncbi_67149	1.14408160400437	6.70762397768944e-29	Nkain1
ncbi_214901	-1.389936082	7.03335543873775e-29	Chtf18
ncbi_26909	-1.668781952	8.03542644453006e-29	Exo1
ncbi_70546	-1.013828745	8.08098141684356e-29	Zdhhc2
ncbi_77744	-1.278295344	9.72791871663864e-29	Bora
ncbi_237542	-1.068718075	1.04098496516289e-28	Osbpl8
ncbi_17133	1.36690500360574	1.48231641184162e-28	Maff
ncbi_219072	-1.502939174	1.63239956823676e-28	Haus4
ncbi_20964	1.05502526932555	1.90777247240482e-28	Syn1
ncbi_66964	-1.01482277	2.04140456123773e-28	Golt1b
ncbi_71643	-1.324747119	2.09311401546027e-28	Zgrf1
ncbi_72080	-1.801748409	2.28320766651048e-28	Sapcd2
ncbi_399568	-1.502543179	2.47949102115717e-28	C15orf41
ncbi_230996	-1.525568754	2.70421314683804e-28	C1orf159
ncbi_71910	-1.305379735	3.07635704517531e-28	Plpp5
ncbi_214240	2.80089146510492	3.71777900289597e-28	Disp2
ncbi_17289	1.17581688330377	3.84192653829437e-28	Mertk
ncbi_70454	-1.725571996	5.19691670716444e-28	Cenpl
ncbi_623474	-1.675688756	5.37316905314172e-28	Rad54b
ncbi_58809	1.0535232977986	5.91850444626991e-28	Rnase4
ncbi_319701	-1.588095748	7.76813907280318e-28	Fbxo48
ncbi_17357	-1.118313324	1.00697317514816e-27	Marcksl1
ncbi_208084	-1.506072156	1.10945797697047e-27	Pif1
ncbi_75939	-1.779810971	1.13943335994275e-27	C4orf46
ncbi_53608	1.3436935892216	2.06047067599864e-27	Map3k6
ncbi_73132	1.14706770845393	2.46608594617591e-27	Slc25a16
ncbi_17155	-1.41563713	2.64287802942792e-27	Man1a1
ncbi_93685	1.7451899421708	3.64235868777114e-27	Entpd7
ncbi_17691	1.15394146465715	4.47572406149795e-27	Sik1
ncbi_67384	-1.23026847	4.72186939589769e-27	Bag4
ncbi_230376	-1.1526076	4.96917112996947e-27	HAUS6

ncbi_56811	1.26001670646291	5.69708288270531e-27	Dkk2
ncbi_70645	-2.048597357	7.98677661112984e-27	Oip5
ncbi_66578	-1.582629525	1.57328023274243e-26	Mis18a
ncbi_58186	-1.109795929	2.82766030341496e-26	Rad18
ncbi_18569	1.21766485279947	3.64874152558162e-26	Pdcd4
ncbi_210035	-1.076668236	5.79407451174499e-26	Nemp1
ncbi_217946	-1.140705815	6.49794190593014e-26	Cdca7l
ncbi_66140	-1.248818597	6.61493412811141e-26	Ska2
ncbi_230098	-1.840788682	7.06066624257313e-26	Arhgef39
ncbi_69270	-2.217383516	7.25771290782337e-26	Gins1
ncbi_208691	-1.32698903	8.61337853214938e-26	EIF5A2
ncbi_18120	-1.190829573	8.74652424617854e-26	Mrpl49
ncbi_233876	-1.065253962	1.1533545072489e-25	Hirip3
ncbi_16651	-1.202962132	1.18562613983047e-25	Sspn
ncbi_641340	1.46388329665319	1.75279892999806e-25	Nrbf2
ncbi_74030	1.03258867377816	2.00168602061786e-25	Rin2
ncbi_69639	-1.122105995	2.12828441219332e-25	Exosc8
ncbi_217340	-1.132614211	2.14374107844904e-25	Rnf157
ncbi_72657	-1.28276117	2.31523293430677e-25	-
ncbi_76478	-1.529187394	3.54905391904143e-25	Haus8
ncbi_12227	1.19866090122068	3.8721859316779e-25	Btg2
ncbi_231506	-1.099724554	4.33049529159843e-25	Lin54
ncbi_320705	-1.113042925	5.29429320524421e-25	Bend6
ncbi_76498	-1.691189228	6.35102031413332e-25	Paqr4
ncbi_233064	-1.178548089	8.45620504967062e-25	Wdr62
ncbi_26570	-1.587038321	9.5277394510456e-25	Slc7a11
ncbi_240055	-1.506558462	1.02904344366024e-24	Neurl1b
ncbi_66934	-1.247652567	1.23597622169054e-24	Dsn1
ncbi_13555	-1.122491527	5.23139508287582e-24	E2f1
ncbi_71323	1.03975640418409	6.36024342327547e-24	Rassf8
ncbi_71924	-1.871589741	7.50145089807072e-24	Tube1
ncbi_50915	2.05683122259668	8.07364820961562e-24	Grb14
ncbi_545428	1.13676832854319	8.241278120275e-24	CCDC141
ncbi_16663	-1.10872574	8.61809512383464e-24	Krt13
ncbi_171285	1.31462524040289	9.27031026300029e-24	Havcr2
ncbi_70382	-1.364882639	1.21334825867924e-23	KCTD2
ncbi_320209	-1.300881626	1.28654709235804e-23	Ddx11
ncbi_71151	-1.29675651	2.06691252501761e-23	Eri2
ncbi_76464	-1.694451454	2.11244393512554e-23	Knl1
ncbi_72568	-1.29032767	2.60469767239315e-23	Lin9
ncbi_78558	-1.38736327	2.86995188345109e-23	Htra3
ncbi_12545	-1.362586408	3.14999747153908e-23	Cdc7
ncbi_104184	-1.172274331	4.02492135676025e-23	Blmh
ncbi_15288	-1.143963844	4.14092727383678e-23	Hmbs

ncbi_22036	-1.43452665	4.24570222918151e-23	Traip
ncbi_16180	-1.112428182	5.3985107885088e-23	Il1rap
ncbi_327762	-1.462052766	7.90741496528187e-23	Dna2
ncbi_231050	-1.060992505	1.21060304067785e-22	Galnt11
ncbi_14933	-1.435703862	1.29593377826491e-22	Gk
ncbi_12366	-1.051749682	1.92843306655144e-22	Casp2
ncbi_64899	1.04929689058432	2.14205870093975e-22	Lpin3
ncbi_69922	-1.271201336	2.97612399228884e-22	Vrk2
ncbi_230596	-1.148648774	3.14036893107114e-22	Prpf38a
ncbi_15040	1.35818672114776	4.00907017612013e-22	H2-T23
ncbi_78038	-1.850892723	4.31119836170913e-22	Mccc2
ncbi_94242	1.14413190945012	4.77214427529804e-22	Tinagl1
ncbi_27756	-1.078857025	5.26501674173872e-22	LSM2
ncbi_76044	-1.976891034	5.92959769206309e-22	Ncapg2
ncbi_245944	-1.016855316	6.9639600855863e-22	Vps54
ncbi_72960	1.77119317788809	9.59160888985766e-22	Top1mt
ncbi_243529	-1.148177177	1.18364079494146e-21	H1-10
ncbi_216198	1.99733542500635	1.21175918631215e-21	Tcp11l2
ncbi_15425	1.36826993079074	1.34867938869212e-21	HOXC6
ncbi_23885	-1.066714732	1.37446263803529e-21	Gmcl1
ncbi_15983	-1.253310482	2.01760199383115e-21	IFRD2
ncbi_381199	1.26807294216879	3.92433913048056e-21	Tmem151a
ncbi_108943	-1.289392247	4.97478989419831e-21	Trmt10a
ncbi_65099	-1.741081703	6.00143048973454e-21	Irak1bp1
ncbi_66686	-1.645494196	6.50971395286102e-21	Dcbld1
ncbi_12994	-3.403100921	8.50890031551719e-21	Csn3
ncbi_60411	-1.841211524	9.82628992512206e-21	Cenpk
ncbi_209462	-1.123103104	1.02777887582729e-20	Hace1
ncbi_15116	1.05890462059041	1.09639396911002e-20	Has1
ncbi_14239	1.08853447299239	1.29329403463285e-20	Foxs1
ncbi_54635	1.01439418519996	1.33691886842006e-20	Pdgfc
ncbi_17434	1.33325934404492	1.50165700147341e-20	Mocs2
ncbi_18099	1.02289680353001	2.03181319722187e-20	NLK
ncbi_14211	-1.481025859	2.06212338547315e-20	Smc2
ncbi_72194	1.0681262177055	2.21590116049206e-20	FBXL20
ncbi_192197	1.14014367847133	3.05626926570737e-20	Bcas3
ncbi_102545	-1.394705253	7.00783935354878e-20	Cmtm7
ncbi_76088	1.05525672392622	7.53538270021442e-20	Dock8
ncbi_70088	-1.411630898	1.01961214850831e-19	Meaf6
ncbi_209357	1.22200657656147	1.1341691622733e-19	Gtf2h3
ncbi_18392	-1.782636305	1.20933718139299e-19	Orc1
ncbi_20319	-1.208337401	1.2986551658665e-19	Sfrp2
ncbi_115489888	-1.411817769	1.56330566804701e-19	gag-pol
ncbi_12349	-1.243447438	2.10757223890558e-19	Ca2

ncbi_14858	1.46038730900792	2.74531605796674e-19	Gsta2
ncbi_72107	-2.238043745	3.06689237895657e-19	DSCC1
ncbi_226409	-1.310136118	3.55068203014131e-19	Zranb3
ncbi_20465	-1.071027372	3.89690634552869e-19	Sim2
ncbi_23947	-1.021702185	3.94058504871581e-19	MID2
ncbi_67824	-1.483315051	5.34000622536682e-19	Nmral1
ncbi_319581	-1.70435123	5.35607646239596e-19	Xkr5
ncbi_66953	-1.21110018	5.82858845111237e-19	Cdca7
ncbi_381101	-1.538473643	6.51618618753276e-19	Dnph1
ncbi_20725	-1.385867764	1.03641231908216e-18	Serpinb8
ncbi_207181	1.20336998472696	1.29926063251021e-18	Rbms3
ncbi_13435	-1.452636303	1.3471376102212e-18	Dnmt3a
ncbi_13531	-2.740374515	1.49277798691414e-18	Usp17la
ncbi_12390	-1.008456388	1.68394795378638e-18	Cav2
ncbi_75746	-1.152641096	1.80171977761191e-18	Morc4
ncbi_67263	1.20844091001161	1.81753246346349e-18	Zswim6
ncbi_103268	-1.25945485	1.92817674117937e-18	Cep57l1
ncbi_66126	-1.196869565	2.05714642461442e-18	ELOF1
ncbi_386463	-1.776847163	2.25461171503174e-18	Cdsn
ncbi_78767	-1.925659601	2.27635551131322e-18	Efcab11
ncbi_60364	-1.267571662	2.34184769205805e-18	Donson
ncbi_66614	1.28051436060299	2.52793317744379e-18	Gpatch4
ncbi_99730	1.20979758481805	3.13083118671162e-18	TAF13
ncbi_214642	1.14791845927642	5.08344691177252e-18	CPED1
ncbi_69716	-1.226190299	6.70013211456052e-18	Trip13
ncbi_232223	1.32865789686344	7.92724726791032e-18	-
ncbi_75420	1.00472133913893	7.99448367925343e-18	Secisbp2
ncbi_269702	-1.045124286	8.41990496840954e-18	Mphosph9
ncbi_77578	1.03348546486367	8.43461529356977e-18	Bcl9
ncbi_241627	-1.074933631	8.99475071390662e-18	Wdr76
ncbi_209707	-1.234680648	1.00978271585057e-17	Gm3414
ncbi_69885	-2.466168197	1.13409918601066e-17	Aunip
ncbi_70083	-1.266299485	1.47135725690669e-17	Metrn
ncbi_18045	-1.030858586	1.69938424436411e-17	Nfyb
ncbi_74653	1.22544128151596	2.17508927180502e-17	Pomk
ncbi_102124	-1.353085413	2.49066515352995e-17	Enkd1
ncbi_65103	-1.078959796	2.90602923544887e-17	Arl6ip6
ncbi_223970	-3.173034259	3.08287948449467e-17	Rmi2
ncbi_58801	1.21840164840916	3.65645809989875e-17	Pmaip1
ncbi_72391	-1.579617484	5.13027201135498e-17	Cdkn3
ncbi_72776	-1.332122152	6.26702405491912e-17	Sass6
ncbi_53886	1.15017211326414	7.08983253252463e-17	Cdkl2
ncbi_140917	-1.648087956	7.78350388940582e-17	Dclre1b
ncbi_547253	1.59881714638189	8.04221699401233e-17	Parp14

ncbi_109145	-1.004578455	1.04526225263794e-16	Gins4
ncbi_218885	-1.069309101	1.09284217038238e-16	Oxnad1
ncbi_19183	-1.861133987	1.15579614101904e-16	Psmc3ip
ncbi_26912	-1.104405165	1.20550400937604e-16	Gcat
ncbi_57434	-1.218599382	2.27267993659605e-16	Xrcc2
ncbi_105377	-1.216099707	2.71083629170362e-16	Slf1
ncbi_52589	-1.798598021	3.81055032699566e-16	NCALD
ncbi_13003	1.01914322557122	3.90728408504296e-16	Vcan
ncbi_230099	-1.979127024	4.16840373753987e-16	Ca9
ncbi_16324	1.31636108598222	4.86883684230184e-16	Inhbb
ncbi_71712	-1.254220357	7.83455916060794e-16	Dram1
ncbi_16511	-1.348798318	8.85365732189934e-16	Kcnh2
ncbi_26875	1.65016076662893	1.31764521000086e-15	Pclo
ncbi_104732	-1.227140263	1.39443106793104e-15	Tedc1
ncbi_245902	-1.287091673	1.44440724614962e-15	Ccdc15
ncbi_20193	1.21393897457097	1.72139330426415e-15	S100a1
ncbi_227102	-1.127913739	1.75051353105779e-15	Ormdl1
ncbi_223780	-2.812451503	2.0800120497347e-15	Adm2
ncbi_100042295	1.70174150131545	3.85684934520952e-15	Gsta1
ncbi_66724	-1.120170175	5.86317892445833e-15	Tab3
ncbi_28010	-1.296650257	7.11759031807252e-15	Miip
ncbi_237211	-1.442711195	7.95814601617622e-15	Fancb
ncbi_68708	1.28389921568852	9.81466589364526e-15	Rabl2
ncbi_223774	-1.219888721	1.01159013322188e-14	Alg12
ncbi_71804	-1.772250033	1.16721922322846e-14	Mtfr2
ncbi_74196	1.05456683693016	1.41804420027146e-14	Ttc27
ncbi_69106	1.07387615133319	1.47994387351029e-14	Stoml1
ncbi_13171	1.3028291280634	1.695675537051e-14	Dbt
ncbi_623781	1.23267597517266	1.80672430909992e-14	Gm14137
ncbi_67196	-1.82970756	1.84042591148689e-14	Ube2t
ncbi_66336	-1.542562832	2.0670309166944e-14	Cenpp
ncbi_665211	1.16783930394866	2.20677436770999e-14	Zfp120
ncbi_76832	-1.05171105	2.52322831305065e-14	Hyls1
ncbi_382639	1.65182445511723	2.59328079309719e-14	Zbtb42
ncbi_268345	1.62702987549813	3.93468027103743e-14	Kcnc2
ncbi_234396	-2.056218637	4.10212013103883e-14	Ankle1
ncbi_77220	1.86020238705128	9.39208158228651e-14	Tmem200a
ncbi_59001	-1.480278974	1.01292956266994e-13	POLE3
ncbi_18861	-1.050610355	1.08781589923283e-13	Rsph10b
ncbi_66488	-1.110972218	1.64780715487372e-13	Fam136a
ncbi_17193	-1.664187676	2.02990162020912e-13	Mbd4
ncbi_56323	1.30062252537997	2.22036026004805e-13	Dnajb5
ncbi_71876	-1.398636849	2.88780202143634e-13	Cenpu
ncbi_67678	-1.053112898	4.4581136867573e-13	LSM3

ncbi_109065	-1.118109398	4.69537839511135e-13	Dnaaf2
ncbi_52076	1.05488628091656	4.7039434471927e-13	Tmem38b
ncbi_17777	1.4802012066014	1.91089151686385e-12	Mttp
ncbi_56318	1.26076985864845	2.15737017644952e-12	Acp3
ncbi_18049	1.16191023261349	2.33721778656359e-12	Ngf
ncbi_17345	-1.579077061	2.54300252971935e-12	Mki67
ncbi_68028	-1.301805805	2.57037185184319e-12	Rpl22l1
ncbi_20649	1.03526330153375	3.03262087132577e-12	Sntb1
ncbi_102632	1.03512318506664	3.22301500540852e-12	Acad11
ncbi_78755	-1.101707086	5.85370286739985e-12	Fam122b
ncbi_15551	1.0707750330414	5.96953921027516e-12	Htr1b
ncbi_68203	1.30882708044762	6.02700141600876e-12	Diras2
ncbi_102639653	1.35821780712715	6.53656587706497e-12	Zfp120
ncbi_239096	-1.428047379	8.78778374272452e-12	Cdh24
ncbi_114714	-1.225521428	1.01661690890017e-11	Rad51c
ncbi_235493	1.00175551437691	1.02038143953748e-11	Fam214a
ncbi_64339	-1.255091826	1.02106850228544e-11	Fndc4
ncbi_100859931	-1.169925001	1.02483265242652e-11	Gon7
ncbi_68487	1.34654105070815	1.02994578398942e-11	Tmem140
ncbi_12062	1.37652018969629	1.23103150095279e-11	Bdkrb2
ncbi_241452	-1.153945341	1.49212624571055e-11	Dhrs9
ncbi_69399	-1.028416471	1.77757871339277e-11	C1orf21
ncbi_67636	1.09381668665411	1.80976593282584e-11	Etfrf1
ncbi_70235	-1.267859179	1.97726730142393e-11	Poc1a
ncbi_69068	1.04223378924119	2.07194624012259e-11	Tcim
ncbi_110052	-1.19898783	2.16258078608319e-11	Dek
ncbi_102371	1.75270589727038	2.20462375186956e-11	Myzap
ncbi_66282	1.26050492574596	2.22504373991125e-11	Tma16
ncbi_76915	-1.05982809	2.63375430298294e-11	Mnd1
ncbi_277396	-1.922320636	2.67991645791885e-11	Klhl23
ncbi_14176	1.99874220127962	2.82238847400743e-11	Fgf5
ncbi_107373	-1.190428493	3.67662208358801e-11	Fam111a
ncbi_70099	-1.491788567	3.99153558498406e-11	Smc4
ncbi_14456	1.03547717903487	4.49594817737858e-11	Gas6
ncbi_243083	-1.425231854	5.10090480565499e-11	Tmprss11f
ncbi_16842	1.23903508653902	5.83384940650102e-11	Lef1
ncbi_319801	1.51669828882652	7.11564317398756e-11	Tigar
ncbi_13654	1.29902480738432	7.49303118500058e-11	Egr2
ncbi_17427	-1.322356894	7.82353846441952e-11	Mns1
ncbi_72016	-1.122110793	8.006034983163e-11	Tedc2
ncbi_381678	1.86260896270608	8.62623203244745e-11	Zcwpw1
ncbi_16449	1.10428635751732	1.43816598945385e-10	Jag1
ncbi_27226	1.26851722006915	1.85143327341348e-10	Pla2g7
ncbi_320438	-1.036431324	1.93923091002537e-10	Alg6

ncbi_77864	1.26201668102759	1.97510113622717e-10	--
ncbi_68597	-1.11082618	2.07585953257822e-10	Ccdc167
ncbi_216835	-1.311427368	2.12092451105002e-10	Usp43
ncbi_72267	-1.110136221	2.14224361349708e-10	Lrrc8e
ncbi_666060	1.17299642102714	2.5471883881135e-10	Frmpd1
ncbi_209086	1.08152155744332	3.22927875414712e-10	Samd9l
ncbi_233826	-1.564313557	3.55277610971585e-10	Palb2
ncbi_66634	-1.173266761	4.10429447799922e-10	Mcm8
ncbi_434341	1.37746834883112	5.14204469817752e-10	Nlrc5
ncbi_23892	1.03392735894207	5.24690161693549e-10	Grem1
ncbi_22276	1.20802828492072	6.50822707062787e-10	Uros
ncbi_26886	-1.375967291	7.16061171356687e-10	Cenph
ncbi_68636	1.57751793521587	1.1893467460704e-09	Fahd1
ncbi_72668	-1.793128024	1.20674253677956e-09	Skida1
ncbi_20305	11.0256010032039	1.21992090265787e-09	Ccl6
ncbi_66443	-1.458430095	1.28388526936922e-09	Tnfaip8l1
ncbi_665700	1.59892095831108	1.3726177748888e-09	Hmcn2
ncbi_73284	1.23099489236286	1.51062753139948e-09	Ddit4l
ncbi_68149	-1.065717112	1.58754052674128e-09	Otub2
ncbi_67475	1.03754362902013	1.62202667953562e-09	Ero1b
ncbi_219151	-1.332931868	1.62840791047834e-09	Scara3
ncbi_93888	1.08309440223941	1.86098713536364e-09	PCDHB16
ncbi_263803	-1.051026868	2.33271257808625e-09	Pkn3
ncbi_407786	-1.329753981	2.43386878698414e-09	Taf9b
ncbi_99890	1.1140225439298	2.44072943733908e-09	Prmt6
ncbi_234728	1.08035070298491	2.59489999454458e-09	Cmtr2
ncbi_67790	-1.646352838	2.63887882327497e-09	Rab39b
ncbi_235345	2.01159126997686	2.71908090124208e-09	C11orf88
ncbi_13838	1.04476220075882	2.72692160344487e-09	Epha4
ncbi_22643	-1.100602863	2.88159274099949e-09	ZNF700
ncbi_18442	1.01360753273162	3.05564534874184e-09	P2ry2
ncbi_223650	1.01462393459678	3.88035925840353e-09	Eppk1
ncbi_245350	1.32106414592304	4.41391571626944e-09	UBE2Q2
ncbi_53978	-1.578576058	4.45829394334538e-09	LPAR2
ncbi_67311	-1.242218352	4.79431696570299e-09	Nanp
ncbi_212706	-1.578289849	5.41782314013431e-09	N4bp3
ncbi_69928	-1.494445296	5.88916493601932e-09	Cenps
ncbi_209003	-1.097481759	5.94044986352354e-09	Rbmx2
ncbi_74470	-1.731234022	6.51584716588863e-09	Cep72
ncbi_100504263	1.42860491561746	6.97664147986858e-09	Zfp120
ncbi_102639598	1.42860491561746	6.97664147986858e-09	Zfp120
ncbi_66114	1.23395717296937	6.98969718963475e-09	Dnajc30
ncbi_103711	1.02939469244121	9.30948137414074e-09	Pnpo
ncbi_100036521	-1.128276712	9.7095059249768e-09	UMAD1

ncbi_18037	1.45650932001975	1.01030233309988e-08	Nfkbie
ncbi_93708	1.13248583397307	1.20471152089599e-08	PCDHGC5
ncbi_67161	-1.208879043	1.33156921968677e-08	Sclt1
ncbi_381110	1.27566978733712	1.56508825132757e-08	Rmdn2
ncbi_381269	-1.227413502	1.71139473612666e-08	Mreg
ncbi_240641	-1.290640533	1.81150604735943e-08	Kif20b
ncbi_434175	-2.507666667	2.03693499675389e-08	Ccnb1
ncbi_50996	-1.075111802	2.09934677278198e-08	Pdcd7
ncbi_17384	1.02567173467848	2.63548804517089e-08	Mmp10
ncbi_241547	1.28035488412493	3.1656597695804e-08	Harbi1
ncbi_667214	1.01852537277515	3.40671491331446e-08	Tgtp1
ncbi_20311	-1.015167981	4.04431961447351e-08	Cxcl5
ncbi_58994	2.30566304052458	4.23464063120612e-08	Smpd3
ncbi_234915	1.37479459553117	4.35144565256172e-08	Cep126
ncbi_116847	-1.025699507	4.99873294374427e-08	Prelp
ncbi_11419	1.44787235975897	5.52229882795812e-08	Asic1
ncbi_19041	-1.64002282	5.59282216061438e-08	Ppl
ncbi_71562	-1.0485053	7.24560057247406e-08	Afmid
ncbi_224796	-2.300104091	7.40384016336751e-08	CLIC5
ncbi_67885	1.76893379016985	7.61939030887807e-08	Mtln
ncbi_71970	1.86820265115914	7.62483055404295e-08	ZBED5
ncbi_55950	-1.020888534	8.09462042188538e-08	Bri3
ncbi_75985	1.55827404760275	8.20150316932094e-08	RAB30
ncbi_80982	1.83581410645579	8.55109916502378e-08	Cemip
ncbi_12335	1.33241122729473	9.26830200064861e-08	Capn3
ncbi_332397	-1.65277614	1.04605210531627e-07	Nanos1
ncbi_23954	1.35629065688843	1.3313417047054e-07	Nek3
ncbi_20360	1.06254753519498	1.36949226939849e-07	Sema6c
ncbi_69871	-1.041854996	1.4315451043759e-07	Ppp1r35
ncbi_329575	1.10954659321254	1.5090646987895e-07	Znf431
ncbi_56464	1.91477899952079	1.53873370391089e-07	Ctsf
ncbi_232430	1.36568793690181	1.86395908304488e-07	Crebl2
ncbi_18507	1.25663982086646	2.08714633781095e-07	Pax5
ncbi_72745	1.07574770105349	2.25813734192139e-07	Tmem161b
ncbi_74053	1.73065182	2.56087181903082e-07	Grip1
ncbi_244421	-1.256572728	2.56642157041297e-07	Lonrf1
ncbi_76877	1.33086121965737	2.74145926738368e-07	Rab36
ncbi_67980	1.0339749532677	2.74258457673115e-07	Gnpda2
ncbi_209012	-1.141988429	2.81179217794205e-07	Ulk4
ncbi_75690	1.97212531157653	3.01987654623874e-07	Vsig10l
ncbi_230751	1.23728828139002	3.05705400600332e-07	Oscp1
ncbi_93891	1.26568099311824	3.35149336590212e-07	PCDHB14
ncbi_12351	10.2384047393251	4.0202582441481e-07	Ca4
ncbi_60613	1.35195603907097	4.73905798872768e-07	Kcnq4

ncbi_70603	-1.442359758	5.07139329838201e-07	Mutyh
ncbi_68209	-1.195828106	6.4788010906083e-07	Rnaseh2c
ncbi_268297	1.42839437388347	6.57294381290818e-07	Scml4
ncbi_14659	-1.772196491	7.59629765628177e-07	--
ncbi_329727	1.23231369343337	7.69161554248112e-07	Dennd2c
ncbi_19363	-1.712595356	7.80778578869016e-07	Rad51b
ncbi_13733	8.82230526352358	8.16597215171388e-07	Adgre1
ncbi_18606	2.57315641977198	8.78326450372913e-07	Enpp2
ncbi_73420	-1.155399307	8.83951027913827e-07	Ccsap
ncbi_208111	1.64048409231222	8.9733342097299e-07	Znf431
ncbi_93699	1.51737745138557	9.3783978582403e-07	PCDHGB1
ncbi_242702	1.48464296676471	1.16938822929118e-06	Myom3
ncbi_268465	-1.010788613	1.24242322822242e-06	Eme1
ncbi_626854	1.27991433597755	1.28083037214621e-06	Znf431
ncbi_229841	-1.22243659	1.3488301168441e-06	Cenpe
ncbi_14173	1.03497273754649	1.40732317126562e-06	Fgf2
ncbi_70325	1.1517680694092	1.50526483071204e-06	Pigw
ncbi_16578	1.99139714182574	1.52002048514762e-06	Kif9
ncbi_99681	3.25930582141163	1.61785135382705e-06	TCHH
ncbi_72401	-1.323337862	1.6882340099193e-06	Slc43a1
ncbi_14658	-1.238311976	1.6957448861289e-06	Glrb
ncbi_74552	1.25583890442959	1.88238490752512e-06	Nipal3
ncbi_72562	-1.96208467	2.03043225392519e-06	Pcbd2
ncbi_102640003	-2.890264277	2.13257777772102e-06	C2orf16
ncbi_69706	-1.697897303	2.29573618269291e-06	LRR1
ncbi_16774	1.69254749364034	2.77575558607925e-06	Lama3
ncbi_73166	-1.186786424	2.77829261833351e-06	Tm7sf2
ncbi_93885	1.6007017321909	2.86803802184234e-06	Pcdhb14
ncbi_108000	-1.160526594	3.14967712010111e-06	CENPF
ncbi_74645	-1.375086031	3.67795057800719e-06	Tent5c
ncbi_100042314	2.20357601318953	3.87327998694963e-06	Gsta2
ncbi_73086	-1.371265097	4.158330616781e-06	Rps6ka5
ncbi_320207	1.52989111822748	4.22292020947424e-06	Pik3r5
ncbi_329910	-1.629336239	4.27370920383075e-06	Acot11
ncbi_74186	1.89470328806669	4.48071999744681e-06	Ccdc3
ncbi_74762	-1.17510381	4.52731545501815e-06	Mdga1
ncbi_278279	1.39882485393315	4.59728885706532e-06	Tmtc2
ncbi_76380	-1.437971479	4.78298382522724e-06	Cep112
ncbi_117600	1.65479108127673	4.9003130888428e-06	SRGAP1
ncbi_22341	1.14350268011453	5.97004708393061e-06	Vegfc
ncbi_68067	-1.078177348	6.29406021707826e-06	Mrnip
ncbi_109901	1.05931066800539	6.36935490882533e-06	Cela1
ncbi_227094	-1.040315861	6.72376713505089e-06	Nemp2
ncbi_22417	1.41222402079272	7.35752317848287e-06	Wnt4

ncbi_212114	1.07477261840841	7.79045829583032e-06	Nhlrc3
ncbi_239408	-1.170321587	8.35609345947768e-06	Tmem74
ncbi_12404	-1.230724515	8.52263327563346e-06	Cbln1
ncbi_170776	-3.275153336	8.82993095595066e-06	Cd209c
ncbi_22041	-3.105817649	8.97095064161145e-06	Tf
ncbi_58522	1.17432823670486	9.87097982851453e-06	Trim54
ncbi_75597	-1.323152175	1.01679267041747e-05	Ndufaf2
ncbi_67138	-1.053378725	1.0578457057241e-05	Herc6
ncbi_94040	-1.144253594	1.12031563244743e-05	Clmn
ncbi_353346	-5.336878436	1.31658269082888e-05	Gpr141
ncbi_16181	1.17304723647951	1.34914444798498e-05	Il1rn
ncbi_15220	1.15777146173781	1.44339221405736e-05	Foxq1
ncbi_18126	-2.67556505	1.52517558560202e-05	Nos2
ncbi_115486031	1.31584846077065	1.55189123027498e-05	Znf431
ncbi_15957	1.61581686405881	1.57818741762926e-05	Ifit1
ncbi_72275	1.40827400163352	1.61001431283233e-05	--
ncbi_12715	1.73844287702086	1.66256946026485e-05	Ckm
ncbi_68498	1.07374456000256	1.72948793341041e-05	Tspan11
ncbi_66277	-3.200720088	1.92131683650411e-05	Klf15
ncbi_19091	2.04508788952854	2.06239940746182e-05	Prkg1
ncbi_69757	-1.000308895	2.14808234658272e-05	Leng1
ncbi_20528	-3.232824895	2.22743493269133e-05	Slc2a4
ncbi_100141474	1.36623132150419	2.34945524434988e-05	--
ncbi_79565	1.07033718951149	2.37902996182936e-05	METTL27
ncbi_24014	1.12052018881376	2.38545319878229e-05	Rnasel
ncbi_19259	1.59763542563268	2.38644629913669e-05	Ptpn5
ncbi_16517	-1.360370204	2.44733856589934e-05	Kcnj16
ncbi_319713	1.28218799891109	2.56538802131983e-05	Ablim3
ncbi_18133	1.23896803345297	2.59911717898461e-05	Ccn3
ncbi_68332	1.27422268975823	2.60861234822094e-05	Sdhaf1
ncbi_16409	4.09351188592314	2.6956651041462e-05	Itgam
ncbi_629242	1.03313374383417	2.78990792697554e-05	env
ncbi_66153	1.12057534046519	2.80478444326765e-05	Fbxo36
ncbi_93892	1.09542412695302	3.01472504772587e-05	PCDHB18
ncbi_675812	1.03970419003505	3.01837989963551e-05	ZNF605
ncbi_234912	1.43263261460454	3.04579160039141e-05	Cfap300
ncbi_140477	-1.54303182	3.34757644285032e-05	Dmbx1
ncbi_271711	1.11140327698444	3.36613925628499e-05	Tmem169
ncbi_22256	-1.6808608	3.37219668349348e-05	Ung
ncbi_236920	-1.288179304	3.50792683586141e-05	Stard8
ncbi_16870	-1.96608894	4.2780453200263e-05	Lhx2
ncbi_12180	-1.030499974	4.31624880330595e-05	Smyd1
ncbi_330959	1.08132283143674	4.58798582330964e-05	Snapc5
ncbi_94279	-1.333747475	4.92850739653476e-05	Sfxn2

ncbi_72309	1.03553403383078	5.35536987803415e-05	Tmem158
ncbi_320226	1.29043979642603	6.13673695331409e-05	Ccdc171
ncbi_239337	1.5252936751953	6.51298847870098e-05	Adamts12
ncbi_20491	2.56868696100833	6.9253587377764e-05	Sla
ncbi_235386	1.56360862276077	7.01717697190256e-05	Hykk
ncbi_104080	-2.175195814	7.65565674581822e-05	Nxph4
ncbi_74411	1.04783748590155	7.71556906290725e-05	Plpp6
ncbi_239017	1.26458332083838	8.58225159071458e-05	OGDHL
ncbi_16664	1.2384574558629	9.40191880832835e-05	Krt14
ncbi_13972	-1.103750862	9.50966229527559e-05	Gnb1l
ncbi_11601	1.1812258436555	9.66626543196428e-05	Angpt2
ncbi_17749	-1.029087579	9.67556232332065e-05	Polr2k
ncbi_67252	1.32639779159025	9.83950072904719e-05	Cap2
ncbi_100503583	-1.37665901	0.000100223202260438	Fsbp
ncbi_74574	1.45659445933377	0.000104191336354257	LVRN
ncbi_73914	1.23663695142726	0.000108786658249819	Irak3
ncbi_15372	-1.535763398	0.000115457383646404	Hmx2
ncbi_225362	1.20707447593873	0.000122950393663491	Reep2
ncbi_19713	1.59436119872341	0.000123466726322209	Ret
ncbi_545370	1.33857287788359	0.000126264585126745	Hmcn1
ncbi_16196	1.11189961114673	0.000127287720357595	Il7
ncbi_57444	-1.233890529	0.000128915834222274	Isg20
ncbi_381204	-4.169925001	0.000136074679872141	Naaladl1
ncbi_252903	-1.096632471	0.000154938787137328	Ap1s3
ncbi_54525	3.0782312026637	0.00015629610398933	Syt7
ncbi_107197	1.36102107870441	0.000164070123815874	Uqcc3
ncbi_545391	1.43501722871106	0.000164988406675247	Catspere
ncbi_20609	8.04439411935845	0.000177151656543639	Sstr5
ncbi_14067	2.59626009205555	0.000180677294284372	F5
ncbi_69195	-1.081378396	0.000182518378299617	Tmem121
ncbi_70363	-1.720584453	0.000184112270029653	Fam135b
ncbi_26568	-1.030607337	0.000186516838227643	Slc27a3
ncbi_71911	1.27089877928219	0.000197805849372639	Bdh1
ncbi_73047	-1.094213825	0.000213759659300239	Camk2n2
ncbi_319482	3.10035052533247	0.0002144961418257	FCGBP
ncbi_330463	1.54962942760888	0.000244587262094327	ZNF471
ncbi_231842	2.66717726400934	0.000247348233593053	Amz1
ncbi_93717	1.37236158179455	0.000268339696248311	PCDHGA9
ncbi_12287	1.07832018128383	0.000280056823336904	Cacna1b
ncbi_434179	1.3392670498798	0.00028832002754172	Znf431
ncbi_12362	1.3823895637335	0.000312642755798817	Casp1
ncbi_14998	1.05383097632496	0.000319314530564158	H2-DMa
ncbi_12771	7.55458885167764	0.000390992832734656	Ccr3
ncbi_20276	1.73820876101684	0.000404158969527004	Scnn1a

ncbi_13116	-1.87407756	0.000407373516061649	Cyp46a1
ncbi_68567	1.33190687131941	0.000425039760710748	Cgref1
ncbi_93886	1.52242103465244	0.00042973395055527	PCDHB7
ncbi_16173	1.08867902260024	0.000466810958062986	Il18
ncbi_12353	-1.573692412	0.000477952104946488	Ca6
ncbi_20754	3.75307786107441	0.000490373008366432	--
ncbi_72121	-2.024640453	0.000497849456535249	Dennd2d
ncbi_442827	2.47361752488061	0.000513226661988487	Rab44
ncbi_17082	1.82597060022495	0.000528761792146085	Il1rl1
ncbi_105855	-1.127716856	0.000557215461539766	Nckap1l
ncbi_67432	1.40815708500226	0.00061506218387873	Hoga1
ncbi_67900	-1.019456831	0.000646161681134847	Mtfp1
ncbi_235312	1.33936906809876	0.000649740804001352	C1qtnf5
ncbi_69539	1.92295896423617	0.000649982290431172	Trnp1
ncbi_546143	1.28785499132383	0.000676918745439455	C15orf65
ncbi_625530	-2.502230046	0.00071546123699851	Usp17le
ncbi_102638847	1.578242334	0.00071570646110208	Znf431
ncbi_103967	1.16717439042628	0.000751243122275768	Dnm3
ncbi_23863	1.2308042779573	0.000761493386979382	Dand5
ncbi_73327	-1.181480524	0.000782282794094792	Pradc1
ncbi_57745	1.7184988455074	0.000797186530195202	Znf112
ncbi_227615	1.18944813560077	0.000845816053143523	Tmem203
ncbi_407800	1.22486815158566	0.000851209400005107	Ecm2
ncbi_239650	5.23477614579228	0.000863156514448988	Ccdc184
ncbi_67525	1.04909215418067	0.000870239266430151	Trim62
ncbi_319767	4.4490400127907	0.00090318949778933	ATP10B
ncbi_22439	-1.619065153	0.000968663235924482	Xk
ncbi_74354	1.50348843951466	0.00101035055568045	Lrguk
ncbi_243963	-1.778672167	0.00104626554226674	Znf473
ncbi_545260	-1.781086856	0.00104902314134963	Arsi
ncbi_109594	-1.895571994	0.00106741410539975	Lmo1
ncbi_18619	-1.406308981	0.00115994904269038	Penk
ncbi_242642	1.6786234760447	0.00116648431992477	Hpdl
ncbi_72446	-1.409165282	0.00120556466535078	Prr5l
ncbi_16515	1.70963642106857	0.00122654539222474	Kcnj12
ncbi_230824	2.35373978024753	0.00123798487653376	Grhl3
ncbi_381983	2.11657557684938	0.00123894347330987	Lmtk3
ncbi_242894	1.22239242133644	0.00126405682197323	Actr3b
ncbi_64176	1.45575375570294	0.00129116907197514	Sv2b
ncbi_216441	1.74164621925688	0.00133291547581127	Slc26a10
ncbi_208595	1.04944298656388	0.0013358184322057	Mterf1b
ncbi_224090	1.10395096403891	0.00134020311467985	TMEM44
ncbi_68312	1.46996439828018	0.00146947637305622	Gstm7
ncbi_100534287	-1.276544692	0.00148010718246418	DCHS2

ncbi_104010	-7.026062297	0.00152654998439304	Cdh22
ncbi_80981	1.29878324554091	0.0015642594923266	Arl4d
ncbi_14621	1.20517412287747	0.00156784616629265	Gjb4
ncbi_12794	-1.668501868	0.00180405891202797	CNIH2
ncbi_65086	-1.488451346	0.00183083904797796	Lpar3
ncbi_170721	-6.470319935	0.0019156831732079	Papln
ncbi_213522	1.57300639604239	0.00204537480412297	Plekhg6
ncbi_235180	-1.26411202	0.00205117500421295	Fez1
ncbi_18726	7.70735913208088	0.00213980975270576	Lilrb3
ncbi_68507	1.1586058282693	0.00217946925912052	Ppfia4
ncbi_74471	2.00262070015914	0.00220040983487748	--
ncbi_60533	1.88710594359407	0.00226144864255361	Cd274
ncbi_26970	1.6812259490203	0.00255892391322466	Pla2g2e
ncbi_223864	1.07919465781286	0.00256784658007905	Rapgef3
ncbi_100040298	-1.014391288	0.00259705654566023	RPS8
ncbi_58251	1.40397875017313	0.00260312522393934	Cep295nl
ncbi_207182	1.12373152946109	0.00269289289676582	Ggt7
ncbi_67574	-1.023323522	0.00276943314651258	Alg13
ncbi_78317	1.80302466855887	0.00277255640246161	Ccdc88b
ncbi_269378	-1.196045341	0.00278366368140881	Ahcy
ncbi_22351	-1.038340773	0.00279710920881153	Vill
ncbi_75860	1.53986179640639	0.00284497090222244	Tex26
ncbi_78267	1.20457372226043	0.00302483558616575	Klhdc8b
ncbi_237397	-4.183221824	0.00322589481971511	C2cd4c
ncbi_12768	2.88105892727649	0.00330713810983608	Ccr1
ncbi_241113	1.1774902785625	0.0034557288601662	Prkag3
ncbi_626870	1.77856294260872	0.00346188646998924	Gm11992
ncbi_544881	-7.736965594	0.00348433334385529	Eif1a
ncbi_238875	7.69812585230285	0.00352963442234829	Gapt
ncbi_66412	1.632540345	0.00353489529701814	Arrdc4
ncbi_18826	3.03294409861491	0.00355776722430436	Lcp1
ncbi_75530	1.5220351669443	0.00357024984989014	Lyrm7
ncbi_19073	1.83578916701904	0.00357678074505585	Srgn
ncbi_216350	-1.955639177	0.00369606826596532	Tspan8
ncbi_225392	2.30140577101402	0.00381595646958527	Rell2
ncbi_319924	1.09364815900317	0.00386635359433144	Apba1
ncbi_100043805	-1.852890567	0.00398312669271926	RPL15
ncbi_271424	1.18850508359295	0.00408060430129742	Ip6k3
ncbi_81877	1.63139807371144	0.00448344578152274	TNXB
ncbi_68070	1.21469487156234	0.00454508210923967	Pdzd2
ncbi_268480	1.02703925275235	0.00456594984497273	Rapgefl1
ncbi_66311	-1.203963099	0.00459218859690149	Cenpw
ncbi_100328588	-3.553053253	0.00479393998339316	Il4i1
ncbi_18232	7.35461671359354	0.0048444668201716	Nxph2

ncbi_207474	-1.125174723	0.0049294439967323	KCTD12
ncbi_100303744	-5.252665432	0.0049859869164833	--
ncbi_22249	1.73999328686377	0.00503817311097648	Unc13b
ncbi_118568304	-2.373305454	0.00508276969396808	gag
ncbi_108079	1.00509966964684	0.00509581126977719	Prkaa2
ncbi_20351	1.17302868505071	0.0051190763961504	Sema4a
ncbi_633640	1.11590398714377	0.00525350064733055	Tmem267
ncbi_16191	4.72602008760449	0.00526473861961556	Il5
ncbi_54396	1.30848673951359	0.00528500393663729	Irgm
ncbi_328967	1.43692061717729	0.00538627632654998	Arhgef37
ncbi_14395	6.6724253419715	0.0055198699392306	Gabra2
ncbi_23972	-1.196320736	0.0057527769155017	Papss2
ncbi_18383	1.60825045561506	0.00575901528349316	Tnfrsf11b
ncbi_68917	1.22164785256132	0.00595630914044874	Hint2
ncbi_69553	-1.914379574	0.00601101661919569	Ripor3
ncbi_50782	1.70945403059764	0.00610496233331743	Rgs11
ncbi_67448	1.12852837961115	0.00627300753002413	Plxdc2
ncbi_102626	1.51864824668566	0.00629208281772018	Mapkapk3
ncbi_213391	-1.077675185	0.00638473967464415	Rassf4
ncbi_243743	1.6253202383373	0.00644833650882373	Plxna4
ncbi_17952	1.04972127840821	0.0064938070166574	Naip6
ncbi_17951	2.55095360855626	0.00657701032523381	Naip5
ncbi_72556	1.06234147528649	0.00660312779339244	ZNF566
ncbi_210741	1.88913941065043	0.00661069447441512	Kcnk12
ncbi_12614	1.52580771402126	0.0066159256086117	Celsr1
ncbi_18158	1.14458800463368	0.00674543291261641	Nppb
ncbi_71213	1.23683659720209	0.00712269526143867	Cage1
ncbi_545649	1.58839068857004	0.00712269526143867	Ifna13
ncbi_239849	1.82499508119333	0.00715711178755058	Cd200r4
ncbi_105244828	2.65269389232853	0.00729341617089594	--
ncbi_382083	1.26040702386397	0.00730740981406332	SNX22
ncbi_319446	1.6040528217671	0.00750002598847081	Dpep2
ncbi_108105	-1.531691706	0.00756015511868389	B3gnt5
ncbi_170722	-1.695638732	0.00763787582204904	NXF2
ncbi_381546	-1.102383145	0.00769283505850153	CCDC24
ncbi_331416	1.69441077457407	0.00807166387166295	SYCP3
ncbi_80861	1.29765908012422	0.00808520039280182	Dhx58
ncbi_54123	1.26681761216666	0.00832869867675017	Irf7
ncbi_24084	2.5898447221009	0.00833019141353466	Tekt2
ncbi_77505	1.26130766111433	0.00848161233244169	DNHD1
ncbi_634731	1.00680196941708	0.00865849328834798	SUSD1
ncbi_100039781	1.40297908246836	0.00902713789159557	Hrct1
ncbi_58229	1.23349013021978	0.00917169770568195	Efcc1
ncbi_108167434	-2.742778118	0.00920562504208509	gag-pol

ncbi_75304	1.80859844608881	0.00923882806372794	--
ncbi_16500	2.3109985347291	0.00938649212337151	Kcnb1
ncbi_231986	-2.255124724	0.00956999084762086	Jazf1
ncbi_51938	1.7988131857918	0.00958439488496162	Ccdc39
ncbi_100043772	-1.016354613	0.00966170824274059	Zfp60
ncbi_13076	1.05966292129834	0.00974884359055338	Cyp1a1
ncbi_75051	1.15161745318385	0.0097529628002122	Ccdc173
ncbi_18617	2.50093372614906	0.0109567963960042	Rhox5
ncbi_20765	1.77517363333454	0.0113896682617847	--
ncbi_209176	2.35795640407158	0.0115208701355733	Ido2
ncbi_81879	-2.929921564	0.0118108817504622	Tfcp2l1
ncbi_12560	-4.274480932	0.0119437061857065	Cdh3
ncbi_653016	1.18470749082268	0.0120226390376316	Mymx
ncbi_50786	3.7162070339994	0.0122977873293237	Hs6st2
ncbi_791415	2.66252348401481	0.0123960446121446	--
ncbi_11484	1.31865083717879	0.0124295096062008	Aspa
ncbi_17392	2.74357901162429	0.0124516372414886	Mmp3
ncbi_93883	1.77031754273307	0.012466792417626	Pcdh3
ncbi_69707	1.12360251934047	0.0129621701699622	Iqcg
ncbi_327978	1.00472756853698	0.0129756334895574	Slfn5
ncbi_623230	-1.712281331	0.0132167265617524	TMEM200B
ncbi_192663	-1.472852134	0.0132394444372039	ABCG4
ncbi_65221	2.79986816399558	0.013277543034198	Slc15a3
ncbi_21802	1.31625934480067	0.013619852656779	Tgfa
ncbi_70450	-1.412416119	0.0138018765759328	Unc13d
ncbi_80720	1.86906913017356	0.0138579632793301	Pbx4
ncbi_76872	1.41004719668683	0.0140081464728964	Ccdc116
ncbi_14069	1.14932031093284	0.0141156002558035	F8
ncbi_22634	-1.914270126	0.014403083659632	PLAGL1
ncbi_56219	-1.231922185	0.0148991626472761	Extl1
ncbi_14942	-1.374691449	0.0150097462578979	Gzme
ncbi_399603	-1.206242681	0.015152180573332	LRATD2
ncbi_19130	1.51180938838128	0.0151732370440051	Prox1
ncbi_100271882	1.86782244357034	0.0153107864160579	Zfp120
ncbi_231724	1.44660395843375	0.0154295547682451	Rad9b
ncbi_72544	-1.068090326	0.0157876964860904	Exosc6
ncbi_14706	1.8247768103009	0.0159657056143374	Gng4
ncbi_399599	3.68684211474037	0.0164601416656754	Ccdc87
ncbi_15267	3.15123782496303	0.0164766097118195	H2AC18
ncbi_13024	1.22384821867581	0.016814337368233	Ctla2a
ncbi_100043257	-1.127272996	0.0168145738016168	Rbm3
ncbi_268902	1.20553020414908	0.0174036089455918	Robo2
ncbi_105244717	-3.829584869	0.0174322215646626	-
ncbi_110876	3.49749965947082	0.017549828088366	Scn2a

ncbi_626832	1.09862580663676	0.0177869215242111	Znf431
ncbi_101401	-1.599550037	0.0178300660246772	ADAMTS9
ncbi_19332	-1.126590674	0.0181863269671322	Rab20
ncbi_20375	7.71195370615814	0.018206237302416	Spi1
ncbi_109620	1.78970705623818	0.0182373524714375	Dsp
ncbi_14960	8.06967352780681	0.018586543877596	H2-Aa
ncbi_75732	1.95329361848827	0.0185936927016821	Iqcd
ncbi_241528	2.62894019778176	0.0187486219651642	Lrrc55
ncbi_14728	3.61761883028462	0.019550964360525	Lilrb4
ncbi_16819	-1.785669733	0.0202809518251002	Lcn2
ncbi_242274	-1.126007885	0.0204363803993107	LRRC7
ncbi_76884	1.06923908263143	0.020662539700136	CYFIP2
ncbi_240047	2.63017507760476	0.0209255781155647	Mmp25
ncbi_66337	1.40651011225698	0.0214024117033893	Fam229b
ncbi_107849	1.07619431821625	0.0216227870223096	Prl2c5
ncbi_214384	-2.841302254	0.0217022677217189	Myocd
ncbi_66300	1.29689154395004	0.02173789887781	INAFM1
ncbi_107934	2.37233304832461	0.0219414261512133	Celsr3
ncbi_237553	-1.547065893	0.0219630590837727	Trhde
ncbi_329540	-1.213290214	0.0219775591639341	Nol4l
ncbi_233046	2.14383577257365	0.0224131398337876	Rasgrp4
ncbi_63872	1.54584314965368	0.0227216442475418	Znf296
ncbi_100039052	-1.158910181	0.0227422482977665	Eif1a
ncbi_171211	2.5401082649369	0.0232148255046264	Edaradd
ncbi_53883	-5.115477217	0.0236824028113925	Celsr2
ncbi_19354	4.16262020081908	0.0237386956393061	Rac2
ncbi_19264	3.19033121210415	0.0237392459319565	Ptprc
ncbi_620695	-1.07652674	0.0242077544751168	Ag2
ncbi_58222	6.93073733756289	0.0254112760205995	Rab37
ncbi_13841	1.3866450583689	0.0260647687069596	Epha7
ncbi_225609	1.49983468700577	0.0262125655199822	--
ncbi_338350	1.14657235428823	0.0265777771083825	Acad10
ncbi_12291	-2.616258631	0.0267766973810913	CACNA1G
ncbi_381218	3.04064198449734	0.0275584224164471	Spata6l
ncbi_217082	2.08746284125033	0.0276240994929284	Hlf
ncbi_258967	4.07562770481337	0.0277497438874236	OR4A5
ncbi_72054	7.34281546136118	0.0282919917026046	Cyp4f3
ncbi_73942	1.92657893053063	0.0283122207217507	FAM151B
ncbi_72514	1.115987951	0.0284228943793545	Fgfbp3
ncbi_20965	2.29564140336709	0.029252154737973	Syn2
ncbi_381677	2.31047157945236	0.0294712220521504	Vgf
ncbi_278795	1.13680757822708	0.0297106356994566	LRRC10B
ncbi_268932	-1.916904384	0.0299286067335987	Caskin1
ncbi_11444	1.16992500144232	0.0300185619086685	Chrnb2

ncbi_74519	7.27301849440642	0.0303041488810336	Cyp2j3
ncbi_118567557	-1.325747557	0.0305286633559844	gag-pol
ncbi_100045778	-1.687799537	0.0307460523010885	Rnf223
ncbi_93715	2.54267077900472	0.0310975873665169	PCDHGA7
ncbi_244853	2.86760146449446	0.0315367271080107	Nxpe4
ncbi_22781	2.24954216950568	0.0321712998340133	Ikzf4
ncbi_15567	3.01264420925222	0.032323410399126	Slc6a4
ncbi_433619	2.56050945699662	0.0326189572555648	Kprp
ncbi_278240	2.20163386116965	0.0333717936905669	Spin2c
ncbi_241289	-1.558267634	0.0337010685096854	Ppp1r26
ncbi_240168	-1.575790855	0.0338061860361954	RASGRP3
ncbi_69358	1.05483247603025	0.0338826097611607	Lrrc51
ncbi_75533	2.08204731296604	0.0340118559451427	Nme5
ncbi_268527	1.01945236266618	0.0344819718865032	Greb1
ncbi_118568072	1.14285896622384	0.0350974241294869	Znf431
ncbi_14727	7.61960864352805	0.0353307587790023	Gp49a
ncbi_16175	3.9893527558005	0.0355138235004549	Il1a
ncbi_71564	-2.213354365	0.0361441439337187	Izumo4
ncbi_319530	2.16766549426019	0.0373248980410762	Znf750
ncbi_140781	-1.160633399	0.0373272337713147	Myh7
ncbi_320587	1.24489676773656	0.0373572344938593	Tmem88b
ncbi_73884	4.68182403997375	0.0378278258289615	Zdbf2
ncbi_17202	3.35461671359354	0.0390655437188572	Mc4r
ncbi_72315	-1.658740427	0.0393814219702158	CCDC74A
ncbi_76184	1.71816588703747	0.0401201616408812	Abca6
ncbi_18143	1.49532528823648	0.0407525371958828	Npas2
ncbi_626415	1.19771606748982	0.0409136169698563	--
ncbi_16176	7.55969574211073	0.0410976329482201	Il1b
ncbi_74147	-1.27465761	0.0413190529068241	Ehhadh
ncbi_80797	-1.336831432	0.0420968097089364	Clca3a1
ncbi_17969	2.53007074222508	0.0422775509818369	Ncf1
ncbi_17884	1.30875270613962	0.0423379512821604	Myh4
ncbi_231125	4.60880924267552	0.042929382162327	Zfyve28
ncbi_11554	-1.892494375	0.0432632234732104	Adrb1
ncbi_16792	3.90000421002328	0.0433950884008502	Laptm5
ncbi_71706	-1.102397759	0.0434201854832525	Slc46a3
ncbi_259277	2.2285105304994	0.0434803002515232	Klk8
ncbi_246730	3.84353725836425	0.044200403685991	Oas1a
ncbi_237711	1.13750352374994	0.044531137436678	Eml6
ncbi_69894	1.16835613303462	0.0449000359647119	Fam241b
ncbi_12523	4.33985000288462	0.0450107497426995	Cd84
ncbi_23969	5.88264304936184	0.0458255695322829	Pacsin1
ncbi_19197	8.43879185257826	0.0460878552616983	Pspn
ncbi_104601	2.56902095685214	0.0464543615586268	Mycbpap

ncbi_69453	6.8703647195834	0.0472617677977916	Prss56
ncbi_19883	1.78986749532876	0.0472846856817899	Rora
ncbi_13107	-3.842184906	0.0475124982885048	Cyp2f2
ncbi_14130	3.16992500144231	0.0476948465703839	Fcgr2
ncbi_394432	-1.596029805	0.0480079297315163	Ugt1a7c
ncbi_12801	1.08831980298679	0.0480079297315163	Cnr1
ncbi_380698	1.02466205423427	0.0483455688222116	Obscn
ncbi_19227	1.22683957224464	0.0485342318295106	Pthlh
ncbi_433470	2.25661073788481	0.0485398603517935	Nmes1
ncbi_16633	1.31511756277047	0.0485744805947582	Klra2
ncbi_14675	1.00765357254344	0.0487801275404372	Gna14
ncbi_170748	-1.12641138	0.0495494454330918	--
ncbi_14555	1.28177096841538	0.0495772425756718	Gpd1
ncbi_69743	1.54164507689604	0.0496735593536583	Casz1
